# Remote vs In-home Physician Visits for Hospital-Level Care at Home: A Randomized Clinical Trial

**DOI:** 10.1001/jamanetworkopen.2022.29067

**Published:** 2022-08-30

**Authors:** David M. Levine, Mary Paz, Kimberly Burke, Ryan Beaumont, Robert B. Boxer, Charles A. Morris, Kathryn A. Britton, E. John Orav, Jeffrey L. Schnipper

**Affiliations:** 1 Division of General Internal Medicine and Primary Care, Brigham and Women’s Hospital, Boston, Massachusetts; 2 Harvard Medical School, Boston, Massachusetts; 3 MGH Institute of Health Professions, Boston, Massachusetts; 4 UMass Chan Medical School, Worcester, Massachusetts; 5 Northeastern University Bouvé College of Health Sciences, Boston, Massachusetts; 6 Division of Cardiovascular Medicine, Brigham and Women’s Hospital, Boston, Massachusetts; 7 Department of Biostatistics, Harvard T.H. Chan School of Public Health, Boston, Massachusetts

## Abstract

**Importance:**

Home hospital care is the substitutive provision of home-based acute care services usually associated with a traditional inpatient hospital. Many home hospital models require a physician to see patients at home daily, which may hinder scalability. Whether remote physician visits can safely substitute for most in-home visits is unknown.

**Objective:**

To compare remote and in-home physician care.

**Design, Setting, and Participants:**

This randomized clinical trial assessed 172 adult patients at an academic medical center and community hospital who required hospital-level care for select acute conditions, including infection, heart failure, chronic obstructive pulmonary disease, and asthma, between August 3, 2019, and March 26, 2020; follow-up ended April 26, 2020.

**Interventions:**

All patients received acute care at home, including in-home nurse or paramedic visits, intravenous medications, remote monitoring, and point-of-care testing. Patients were randomized to receive physician care remotely (initial in-home visit followed by daily video visit facilitated by the home hospital nurse) vs in-home care (daily in-home physician visit). In the remote care group, the physician could choose to see the patient at home beyond the first visit if it was felt to be medically necessary.

**Main Outcomes and Measures:**

The primary outcome was the number of adverse events, compared using multivariable Poisson regression at a noninferiority threshold of 10 events per 100 patients. Adverse events included a fall, pressure injury, and delirium. Secondary outcomes included the Picker Patient Experience Questionnaire 15 score (scale of 0-15, with 0 indicating worst patient experience and 15 indicating best patient experience) and 30-day readmission rates.

**Results:**

A total of 172 patients (84 receiving remote care and 88 receiving in-home physician care [control group]) were randomized; enrollment was terminated early because of COVID-19. The mean (SD) age was 69.3 (18.0) years, 97 patients (56.4%) were female, 77 (45.0%) were White, and 42 (24.4%) lived alone. Mean adjusted adverse event count was 6.8 per 100 patients for remote care patients vs 3.9 per 100 patients for control patients, for a difference of 2.8 (95% CI, –3.3 to 8.9), supporting noninferiority. For remote care vs control patients, the mean adjusted Picker Patient Experience Questionnaire 15 score difference was −0.22 (95% CI, −1.00 to 0.56), supporting noninferiority. The mean adjusted 30-day readmission absolute rate difference was 2.28% (95% CI, –3.23% to 7.79%), which was inconclusive. Of patients in the remote group, 16 (19.0%) required in-home visits beyond the first visit.

**Conclusions and Relevance:**

In this study, remote physician visits were noninferior to in-home physician visits during home hospital care for adverse events and patient experience, although in-home physician care was necessary to support many patients receiving remote care. Our findings may allow for a more efficient, scalable home hospital approach but require further research.

**Trial Registration:**

ClinicalTrials.gov Identifier: NCT04080570

## Key Points

**Question:**

When a patient receives acute hospital-level care at home (home hospital), is the use of remote physician visits noninferior to in-home physician visits in terms of safety and patient experience?

**Findings:**

In this 2-site randomized clinical trial of 172 patients, the mean adverse event count was 6.8 per 100 patients for patients receiving remote care vs 3.9 per 100 patients for control patients, for a difference of 2.8, supporting noninferiority, although 19% of patients receiving remote care required in-home physician visits. Patient experience was noninferior.

**Meaning:**

In this study, remote physician visits were noninferior to in-home physician visits during home hospital care for adverse events and patient experience, although in-home physician care was necessary to support 1 in 5 patients receiving remote care.

## Teaser

This randomized clinical trial of adult patients who required hospital-level home care for select acute conditions assesses whether remote physician visits were noninferior to in-home physician visits.

## Introduction

Hospitals are the standard of care for acute illness in the US, but hospital care is expensive and potentially unsafe and uncomfortable, particularly for older individuals.^1,2,3,4^ Home hospital care is the substitutive provision of home-based acute care services usually associated with a traditional inpatient hospital.^5^ Such care is specifically for the acutely ill patient who would have required care in a hospital bed but instead receives that care at home. Prior work^6,7,8,9,10,11,12,13,14,15^ suggests that home hospital care can reduce costs, maintain quality and safety, and improve patient experience for select acutely ill adults who require traditional hospital-level care.

The concept of decentralizing hospital care to the home contrasts with most theories on economies of scale, yet outcomes on cost and quality make the intervention high value.^16,17,18^ One criticism of home hospital care is the inefficiency incurred by the attending physician or advanced practice clinician. Patient-physician ratios are typically lower for home hospital care than traditional medical wards, in part because of the need to travel to each patient. Nurse ratios are usually similar or less per patient compared with traditional medical wards because there is traditionally no nurse coverage at night. In some respects, this intensive, in-home physician approach may prevent the spread and scale of home hospital care, especially to remote rural areas. Whether a physician could substitute all but the first in-home admission visit with remote video visits (but allow for in-home visits if necessary) without negatively affecting safety, readmission, or patient experience is unknown. Because the efficacy of home hospital care has already been well demonstrated,^19,20^ we sought to demonstrate the noninferiority of this hybrid remote physician care model to in-home care. If successful, this program could increase efficiency, catalyze the model’s scale, and open remote physicians to care for acutely ill adults in multiple settings, including dense urban, rural, international, and even in-hospital settings.

## Methods

### Design

We performed a parallel-design, noninferiority randomized clinical trial (RCT) with participants randomized to receive home hospital care with remote physician care (remote) vs usual home hospital care with in-home physician care (control). Patients, study staff, and physicians were not blinded to allocation status. We enrolled participants between August 3, 2019, and March 26, 2020; follow-up ended April 26, 2020. The trial was abruptly halted because of the COVID-19 pandemic, when as much care as possible was shifted to the remote model. The trial protocol was approved by the Mass General Brigham Institutional Review Board. All participants provided written informed consent before randomization. This report follows the Consolidated Standards of Reporting Trials (CONSORT) reporting guideline for randomized studies. The trial protocol can be found in Supplement 1.

### Setting and Participants

Findings from the first home hospital pilot RCT in the US were previously reported^21^ and subsequently replicated in a larger population.^16,22^ In the current study, we generally maintained these methods. Briefly, adult participants were recruited in the emergency department and medical ward at Brigham and Women’s Hospital (an academic medical center) and Brigham and Women’s Faulkner Hospital (a community hospital). Patients were eligible to enroll if they required admission, lived within the 5-mile catchment area, and were acutely ill from a medical condition, such as infection (eg, cellulitis, complicated urinary tract infection, and diverticulitis), heart failure, chronic obstructive pulmonary disease, or asthma. Patients were ineligible if they required critical care, routine administration of controlled substances, an invasive procedure, more than 1 person’s assistance to reach a bedside commode, or advanced imaging, among other criteria.^16^ Patients were not excluded based on insurance status or if they lived alone and were not compensated. We approached all eligible patients. Complete criteria for each diagnosis are in the eAppendix in Supplement 2.

### Randomization and Interventions

Randomization was generated by a biostatistician (E.J.O.) using SAS statistical software, version 9.4 (SAS Institute Inc) and stratified by infection, heart failure, chronic obstructive pulmonary disease or asthma, and other diagnosis, with randomly selected block sizes of 4 or 6 with allocation concealment via sealed, opaque envelopes. Participants who provided informed consent were randomized by trained research study staff to usual home hospital care with an in-home attending physician (control group) or home hospital care with a remote attending physician (remote group).

The previously reported^16^ home hospital intervention method was generally maintained, with the addition of the 2 randomized groups. In the control group, the attending physician performed rounds each day in the patient’s home alongside the home hospital nurse or paramedic. In the remote group, the attending physician performed an initial in-home visit on admission followed by a daily encrypted video visit facilitated by the home hospital nurse or paramedic who was able to perform examination maneuvers as needed, including transmission of heart and lung sounds (Eko Health). Although the default was to see a patient by video each day after an initial in-home visit, the clinical team could choose at any time to see the patient in person if the physician thought it was medically necessary. If a physician’s time on service (typically 7 days) ended but the patient remained on service, the incoming physician would perform a single in-home visit and then continue with remote visits. With the exception of the physician interaction, patients otherwise received the same home hospital services, including twice-daily, in-home nurse visits (Mass General Brigham Home Care), intravenous infusions via programmable pump (Smiths Medical), respiratory therapies, continuous remote monitoring with a wearable patch that transmitted alarms directly to clinician phones (VitalConnect), and point-of-care testing with the iSTAT (Abbott Laboratories). In both groups, patients had immediate after-hours access via video and telephone to their physician, who could dispatch a mobile integrated health paramedic to the home who could then bring the physician in by video as needed for management. Training of the physicians and nurses included a 1-hour didactic session on best practices in telemedicine. Patients and caregivers received minimal training in telemedicine on admission. Physicians were not given specific protocols for patient management, and patients were discharged when deemed to no longer require acute care.

### Outcomes and Follow-up

The study period began on admission and ended 30 days after discharge. For both groups, study staff interviewed patients on admission and after discharge (contact via telephone attempted between 1 day and 30 days after discharge). On admission, patients reported their sociodemographic characteristics and completed assessments of frailty (Program of Research on Integration of Services for the Maintenance of Autonomy [PRISMA-7]),^23^ cognitive impairment (Ascertain Dementia 8-item Informant Questionnaire),^24^ depression (Patient Health Questionnaire 2),^25^ emotional support (Patient-Reported Outcomes Measurement Information System),^26^ health literacy (Brief Health Literacy Screener),^27^ quality of life (EuroQol Visual Analog Scale),^28^ and functional status (activities of daily living and instrumental activities of daily living).^29^ Study staff collected information from clinical staff and the electronic health record (EHR) for all other variables. Race and ethnicity were included as variables in this study because they have been associated with the social determinants of health.

Our primary outcome was the number of adverse events that occurred during a patient’s admission. For interpretability, we report the rate of adverse events per 100 patients. Adverse events included fall, delirium, potentially preventable venous thromboembolism, new pressure injury, thrombophlebitis at peripheral intravenous site, catheter-associated urinary tract infection, new *Clostridioides difficile* infection, new methicillin-resistant *Staphylococcus aureus* infection, new arrhythmia, hypokalemia, acute kidney injury, transfer back to the hospital, unplanned mortality during the admission, and unplanned mortality within 30 days after discharge. These adverse events were drawn from prior home hospital literature and hospital safety literature.^6,30^ All data were extracted through medical record review by trained research assistants (M.P., K.B., and R.B.). When necessary, the research assistant could ask the nurse for clarification.

Our secondary outcomes were the Picker Patient Experience Questionnaire 15 (scale: 0-15, with 0 indicating worst patient experience and 15 indicating best patient experience),^31^ global experience (scale: 0-10, with 0 indicating worst patient experience and 10 indicating best patient experience), and 30-day readmission. Both experience measures were obtained during the postdischarge telephone call. For global experience, patients were asked, “Using any number from 0 to 10, where 0 is the worst hospital possible and 10 is the best hospital possible, what number would you use to rate home hospital during your stay?” If patients were unable to be reached after discharge (37 total patients, including 18 in the control group and 19 in the remote group), we did not measure patient experience. Thirty-day readmission started with discharge from the acute care episode (from home or the hospital in the case of an escalation). For 30-day readmission, in addition to EHR records from all Mass General Brigham facilities (the health care system that includes Brigham and Women’s Hospital and Brigham and Women’s Faulkner Hospital), we used the CareEverywhere system that joins all institutions that use the Epic EHR.

We explored health care utilization during the acute care episode. Utilization included laboratory orders, radiology studies, consultations, and length of stay. All measures were derived from the EHR. We also measured each home visit’s length, defined as time of entry into the home subtracted from time of exit from the home in minutes, reported by the clinician. In the postdischarge period, we additionally measured whether a patient had an emergency department visit (unrelated to readmission) within 30 days or a primary care visit within 14 days.

### Statistical Analysis

During the prior RCT of home hospital,^16^ patients experienced a mean (SD) of 9 (29) adverse events per 100 patients during their home hospital admission. We believe the smallest clinically meaningful treatment difference would be 10 adverse events (ie, from 9 to 19 adverse events per 100 patients). In this noninferiority trial (α = .025, power of 80%, and noninferiority limit of 0.1 events), we required 210 patients (105 in each arm). As noted above, the RCT was stopped early because of the COVID-19 pandemic. Given that we prespecified only 3 secondary outcomes, we did not account for multiple comparisons. We report our nonprimary and nonsecondary outcomes descriptively only.

We followed an intent-to-treat approach and assessed the noninferiority of the primary outcome with a generalized linear model using Poisson regression and a prespecified noninferiority threshold of 10 events per 100 patients (trial protocol in Supplement 1). For our secondary outcomes, we used logistic regression for 30-day readmission (noninferiority threshold of 5%) and a generalized linear model with a γ-distribution and log link for the Picker and global experiences (noninferiority threshold of 2 points for both). When the 95% CI of the difference failed to cross a noninferiority threshold, noninferiority was considered to be upheld. If a 95% CI of the difference crossed zero and the noninferiority margin, it was considered inconclusive (failed to reject that remote care was inferior).^32^ For all models, we prespecified adjustments for age, gender, race and ethnicity, PRISMA-7 (a measure of frailty),^23^ and HOSPITAL (hemoglobin at discharge, discharge from an oncology service, sodium level at discharge, procedure during the index admission, index type of admission, number of admissions during the last 12 months, and length of stay) score (a predictor of potentially avoidable readmission).^33^ Because of missing data (26 controls and 20 remote care patients), we removed PRISMA-7 from the final models, although the randomized design provided good balance between arms for available PRISMA-7 values. We performed 2 post hoc models: one without the HOSPITAL score given it was based on variables known at discharge and another with the addition of primary language; however, neither of these models changed our findings.

We present descriptive data with numbers (percentages), means (95% CIs), or medians (IQRs) as appropriate. We present both unadjusted and adjusted outcomes for our primary and secondary outcomes. We performed all analyses in SAS statistical software, version 9.4 (SAS Institute Inc).

## Results

### Patient Demographic Characteristics

Of the 277 patients who were screened for eligibility, 172 were randomized (84 remote care patients and 88 controls; mean [SD] age, 69.3 [18.0] years; 97 [56.4%] female and 75 [43.6%] male; 33 [19.3%] Black, 39 [22.8%] Latinx, 77 [45.0%] White, and 22 [12.9%] of other race or ethnicity), and all received their allocated treatment (Figure). Patients who were female (70 of 105 [66.7%] vs 97 of 172 [56.4%]) and had Medicaid (14 of 105 [13.3%] vs 7 of 172 [4.1%]) declined more often, whereas patients who were Latinx were enrolled more often (39 of 171 [22.8%] vs 9 of 105 [8.6%]) (eTable 1 in Supplement 2).^34^

**Figure.**
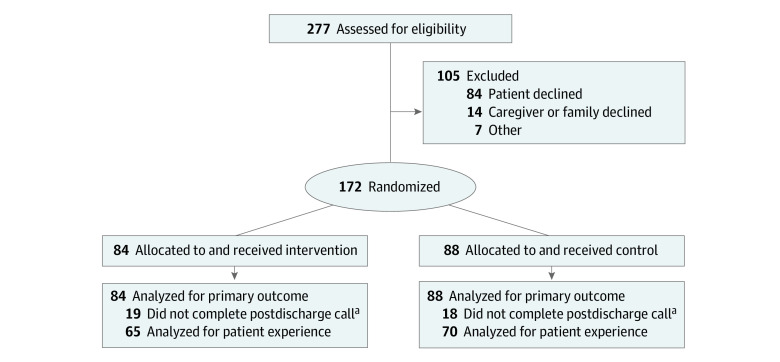
Flow of Participants in the Study ^a^Not completing a postdischarge call incurred missing values for patient experience.

Patients in the control group were younger (mean [SD] age, 66.5 [18.9] vs 72.1 [16.6] years) and more often White (45 [51.7%] vs 32 [38.1%]), privately insured (33 [37.5%] vs 18 [21.4%]), and employed but otherwise clinically similar. Twenty-two patients (26.2%) in the remote care group and 20 (22.7%) in the control group lived alone. At baseline in both groups, patients were frail and chronically ill, had several limitations in their functional status, had frequently used hospital and emergency care in the past 6 months, and had fair health-related quality of life (Table 1). Both groups had similar proportions of patients in the prespecified blocked strata of infection, heart disease, respiratory disease, and other diagnoses. On discharge, the mean HOSPITAL score was 3.0 (95% CI, 2.7-3.4) for the remote group vs 2.7 (95% CI, 2.4-3.1) for the control group.

**Table 1.** Baseline Patient Characteristics^a^

Characteristic	Remote care group (n = 84)	Control group (n = 88)	*P* value
Age, mean (SD), y	72.1 (16.6)	66.5 (18.9)	.04
Sex			
Female	51 (60.7)	46 (52.3)	.28
Male	33 (39.3)	42 (47.7)	
Race or ethnicity			
Black	16 (19.0)	17 (19.5)	.15
Latinx	21 (25.0)	18 (20.7)	
White	32 (38.1)	45 (51.7)	
Other^b^	15 (17.9)	7 (8.0)	
Partner status			
Partnered	35 (41.7)	46 (52.9)	.55
Divorced	9 (10.7)	6 (6.9)	
Widowed	11 (13.1)	7 (8.0)	
Single, never partnered	29 (33.4)	27 (31.0)	
Other	0	1 (1.2)	
Primary language			
English	57 (67.9)	67 (76.1)	.25
Spanish	23 (27.4)	20 (22.7)	
Other	4 (4.8)	1 (1.1)	
Insurance			
Private	18 (21.4)	33 (37.5)	.09
Medicare	53 (63.1)	40 (45.5)	
Medicaid	4 (4.8)	3 (3.4)	
Medicare and Medicaid	9 (10.7)	11 (12.5)	
None	0	1 (1.1)	
Educational level			
Less than high school	18 (28.1)	10 (15.9)	.25
High school	12 (18.8)	18 (28.6)	
<4-y College	15 (23.4)	12 (19.1)	
4-y College	10 (15.6)	16 (25.4)	
>4-y College	9 (14.1)	7 (11.1)	
Employment			
Employed	13 (16.5)	24 (30.4)	.11
Unemployed	14 (17.7)	13 (16.5)	
Retired	52 (65.8)	42 (53.2)	
Cigarette smoking			
Never	42 (50.6)	36 (40.9)	.05
Current	4 (4.8)	14 (15.9)	
Prior	37 (44.6)	38 (43.2)	
Lives alone	22 (26.2)	20 (22.7)	.72
PRISMA-7 score (range, 0-7), mean (95% CI)	3.4 (3.0-3.8)	3.1 (2.7-3.5)	.37
AD-8 score (range, 0-8), mean (95% CI)	1.6 (0.9-2.3)	0.9 (0.5-1.4)	.09
BHLS score (range, 4-20), mean (95% CI)	17.2 (15.9-18.4)	16.9 (15.6-18.2)	.77
No. of chronic comorbidities, mean (95% CI)	3.9 (3.4-4.4)	4.1 (3.6-4.7)	.53
Full code status^c^	71 (85.5)	76 (88.4)	.65
No. of ADLs on admission (range, 0-6), mean (95% CI)	5.0 (4.6-5.5)	5.2 (4.9-5.6)	.49
No. of IADLs on admission (range, 0-8), mean (95% CI)	5.2 (4.6-5.9)	5.4 (4.7-6.1)	.74
PHQ-2 (range, 0-6), mean (95% CI)	1 (0.6-1.5)	1 (0.6-1.5)	.96
Hospital admission in last 6 mo	29 (34.5)	30 (34.1)	>.99
Emergency department visit in last 6 mo	27 (32.1)	40 (45.5)	.09
EQ-VAS score (range, 0-100), mean (95% CI)	55.8 (49.5-62.2)	57.0 (50.8-63.2)	.79
Diagnosis, No. (%)^d^			
Infection			.98
Pneumonia	14 (16.7)	14 (15.9)	
Skin/soft-tissue infection	9 (10.7)	13 (14.8)	
Complicated UTI or pyelonephritis	17 (20.2)	12 (13.6)	
Other infection	11 (13.1)	10 (11.4)	
Heart failure	9 (10.7)	13 (14.8)	
Airway disease			
Asthma	8 (9.5)	8 (9.1)	
COPD	8 (9.5)	10 (11.4)	
Other^e^	8 (9.5)	8 (9.1)	

Abbreviations: AD-8, Ascertain Dementia 8-item Informant Questionnaire (with 0-1 indicating normal cognition and ≥2 indicating cognitive impairment likely to be present); ADLs, activities of daily living; BHLS, Brief Health Literacy Screener (with 4-12 indicating limited; 13-16, marginal; and 17-20, adequate); COPD, chronic obstructive pulmonary disease; EQ-VAS, EuroQol Visual Analog Scale (with 0 indicating worst imaginable health and 100 indicating best imaginable health); IADLs, instrumental activities of daily living; PHQ-2, Patient Health Questionnaire 2 (scale of 0-6, with scores ≥3 indicating possible depression); PRISMA, Program of Research on Integration of Services for the Maintenance of Autonomy (with scores ≥2 indicating frailty); UTI, urinary tract infection.

^a^ Data are presented as number (percentage) of patients unless otherwise indicated.

^b^ Other includes Asian and multiracial.

^c^ Patients who chose resuscitation and intubation.

^d^ Diagnoses were block randomized at the level of infection, heart failure, airway disease, and other.

^e^ Other diagnoses, such as atrial fibrillation with rapid ventricular response, diabetes, pulmonary embolism, and others (see eTable 2 in Supplement 2 for complete list and criteria).

### Adverse Events, Patient Experience, and 30-Day Readmission

Few adverse events occurred. The 3 adverse events were transfer back to the hospital (3.6 per 100 patients for remote care patients and 2.3 for control patients), delirium (4.8 for remote care patients and 1.1 for control patients), and fall (1.2 for remote care patients and 2.3 for control patients) (Table 2). The mean adjusted adverse event count was 6.8 events per 100 patients (95% CI, 2.9-15.7) for remote care patients and 3.9 events per 100 patients (95% CI, 1.4-11.0) for control patients (difference, 2.8; 95% CI, −3.3 to 8.9), supporting noninferiority (Table 3).

**Table 2.** Adverse Events

Adverse event	No. (%) of adverse events	
	Remote care group (n = 84)	Control group (n = 88)
Fall	1 (1.2)	2 (2.3)
Loss of consciousness	0	0
Delirium	4 (4.8)	1 (1.1)
Potentially preventable VTE	0	0
New pressure ulcer	0	0
Thrombophlebitis at peripheral intravenous site	0	0
CAUTI	0	0
New *Clostridioides difficile*	0	0
New MRSA	0	0
New other hospital-acquired infection	0	0
Transfer back to hospital	3 (3.6)	2 (2.3)
Mortality (unplanned) during admission	0	0
Mortality (unplanned) 30-d postdischarge	0	0

Abbreviations: CAUTI, catheter-associated urinary tract infection; MRSA, methicillin-resistant *Staphylococcus aureus*; VTE, venous thromboembolism.

**Table 3.** Noninferiority Analysis: Adverse Events, 30-Day Readmission, and Patient Experience

Measure	Mean (95% CI)		
	Remote care group (n = 84)	Control group (n = 88)	Difference^a^
Primary outcome			
Adverse event count per 100 patients, unadjusted	6.0 (2.5 to 14.3)	3.4 (1.1 to 10.6)	2.5 (–4.0 to 9.1)
Adverse event count per 100 patients, adjusted^b^	6.8 (2.9 to 15.7)	3.9 (1.4 to 11.0)	2.8 (–3.3 to 8.9)
Secondary outcomes			
Picker Patient Experience Questionnaire 15 score, unadjusted^c^	13.49 (12.96 to 14.05)	13.75 (13.23 to 14.29)	–0.26 (–1.03 to 0.50)
Picker Patient Experience Questionnaire 15, adjusted^b^	13.46 (12.93 to 14.01)	13.68 (13.16 to 14.22)	–0.22 (–1.00 to 0.56)
Global experience, unadjusted^d^	9.46 (9.23 to 9.69)	9.67 (9.45 to 9.90)	–0.21 (–0.53 to 0.11)
Global experience, adjusted^b^	9.50 (9.28 to 9.73)	9.62 (9.40 to 9.84)	–0.12 (–0.44 to 0.21)
30-Day readmission rate, unadjusted	8.33 (4.03 to 16.46)	5.68 (2.39 to 12.93)	2.65 (–4.99 to 10.29)
30-Day readmission rate, adjusted^b^	5.70 (2.28 to 13.56)	3.43 (1.11 to 10.07)	2.28 (–3.23 to 7.79)

^a^ Difference represents the remote care group minus the control group.

^b^ Adjusted for age, sex, race or ethnicity, and HOSPITAL (hemoglobin at discharge, discharge from an oncology service, sodium level at discharge, procedure during the index admission, index type of admission, number of admissions during the last 12 months, and length of stay) score.

^c^ Picker Patient Experience Questionnaire 15 is a 0- to 15-point score, with more points indicating a better experience.

^d^ Global experience is a 0- to 10-point scale with 0 indicating the worst hospital possible and 10 indicating the best hospital possible.

The mean adjusted Picker Patient Experience Questionnaire 15 score was 13.46 (95% CI, 12.93-14.01) for remote care patients and 13.68 (95% CI, 13.16-14.22) for control patients (difference, −0.22; 95% CI, −1.00 to 0.56), supporting noninferiority (Table 3). Mean adjusted global experience was noninferior between remote care patients (9.50; 95% CI, 9.28-9.73) and control patients (9.62; 95% CI, 9.40-9.84) (difference, −0.12; 95% CI, −0.44 to 0.21). The mean adjusted 30-day readmission rate was 5.70% (95% CI, 2.28%-13.56%) for remote care patients and 3.43% (95% CI, 1.11%-10.07%) for control patients (difference, 2.28; 95% CI, −3.23 to 7.79), which was inconclusive.

### Utilization and Operational Metrics

Patients were admitted for a median of 4 days (IQR, 3-6 days) in the remote care group and 4 days (IQR, 3-5 days) in the control group (Table 4). Nurse or paramedic visits took somewhat longer in the remote care group (median 46.5 minutes [IQR, 38.6-57.4 minutes]) vs the control group (38.0 minutes [IQR, 31.9-49.0 minutes]). Patient disposition was similar in both groups, with somewhat higher rates of home health disposition in the remote group (7 [8.3%]) compared with the control group (2 [2.3%]). Utilization within the 30-day postacute episode was similar for primary care follow-up and 30-day emergency department presentation.

**Table 4.** Utilization and Operational Metrics

Measure	Remote care group (n = 84)	Control group (n = 88)
During acute episode		
Length of stay, median (IQR), d	4 (3-6)	4 (3-5)
Nurse or paramedic visit duration, median (IQR), min	46.5 (38.6-57.4)	38.0 (31.9-49.0)
Disposition, No. (%)^a^		
Routine	72 (85.7)	83 (94.3)
Home health	7 (8.3)	2 (2.3)
Home hospice	1 (1.2)	1 (1.1)
Other	4 (4.8)	2 (2.3)
30-d Postacute care episode		
Primary care visit within 14 d after discharge, No. (%)	42 (50.0)	40 (45.5)
30-d ED presentation, No. (%)	11 (13.1)	11 (12.5)

Abbreviation: ED, emergency department.

^a^ Disposition following the acute care episode.

### Physician Visits

Each patient received at least 1 physician visit each day, either in the home or remotely. Among patients in the remote care group, 16 (19.0%) required in-home physician visits beyond the initial in-home admission visit. These patients spoke Spanish more often and were able to perform fewer activities and instrumental activities of daily living, although this post hoc analysis was limited by a small sample size (eTable 2 in Supplement 2). Among patients who required an additional in-home physician visit, physicians performed a median of 2.5 (IQR, 1.0-3.0) additional in-home visits per patient.

## Discussion

In this 2-site, noninferiority RCT of home hospital care delivery, we found that patients who received predominantly remote physician care compared with only in-home physician care had a noninferior number of adverse events and noninferior patient experience, although the ability to deliver in-home physician care for remote patients was critical to the care of 1 in 5 patients. Individual programs must decide, however, whether a potential increase in 2.8 adverse events per 100 patients is clinically meaningful to their program and patients.

Home hospital care traditionally has been delivered through in-home care teams with excellent clinical outcomes.^6,15,16^ One known inefficiency of home hospital care is the time required to travel to patients’ homes. Home hospital care models now lie on a spectrum with respect to physician care. On one end, some newer home hospital care models involve complete remote physician care to alleviate the physician’s travel burden, although the safety and efficacy of remote physician evaluation and management for acutely ill patients has been unclear. On the other end, in some models, physicians continue to see all patients in the home. Our model was a hybrid, lying perhaps in the middle of the spectrum, in that it required an initial in-home physician visit, allowed for additional in-home visits if deemed necessary, and depended heavily on a facilitated care interaction between the physician and patient whereby the nurse or paramedic performed examination maneuvers and served as a facilitator throughout the visit.

There are clear trade-offs to the remote physician model. On one hand, physicians have reduced travel burden and are thus able to spend more time selecting and recruiting patients in the hospital and caring for patients in general, increasing their efficiency. This approach could also in the future allow for physicians to cover a higher patient census or larger catchment areas and could facilitate expansion to more rural areas. On the other hand, nurse and paramedic visit times increased 8.5 minutes with remote care. This increase was almost certainly because a second pair of hands makes visits more efficient by allowing for accomplishing tasks in parallel rather than in sequence, helps teams be aligned on the care plan, and uses travel time to discuss patients and coordinate care. In addition, facilitating the physician’s evaluation requires additional time. Whether the value gained from remote physician visits outweighs the 8.5 minutes lost per visit for nurse and paramedic team members is a question each home hospital service should assess locally.

We did not test a fully remote physician model; therefore, our data do not support or refute fully remote physician care. However, it is likely that the remote group’s outcomes depended on and would not have reached noninferiority had these in-home physician visits not occurred. As a result, we believe it is necessary for any home hospital program to have the capacity to provide in-home physician visits. Given the important proportion of patients who required additional in-home physician visits despite randomization to the remote group, a fully remote model likely would underserve a sizable proportion of patients. These patients often did not speak English or had severe visual or auditory impairments, cognitive deficits, or complicated presentations that continued to require in-home physician examination. Although our data are limited when examining these patients’ sociodemographic characteristics, we believe our findings point to the importance of providing high-quality interpretation services that are integrated with the video system to ensure equity for patients who do not speak English as their primary language. It is likely that lack of integration between a telephone interpreter and the video connection resulted in degraded sound quality and required in-home visits for better communication. It is also possible that patients with fewer instrumental activities of daily living are likely to require in-home visits.

When clinicians are not forced into a randomization schema, they likely can tailor remote vs in-home visits based on the patient’s needs. For example, a clinician might choose in-home care when a patient is less stable, has a fluctuating physical condition, has multiple tasks to be completed, speaks a language different from the physician and/or nurse, has sensory impairments that cannot be overcome, or is cognitively impaired. Patients or their family may also have a strong preference for in-home care. In contrast, a patient progressing along the plan of care without large changes in management might be more amenable to remote care. The flexibility of the program design to allow for in-home visits when needed with most of the care delivered in a remote fashion may be most effective. Future work can include risk stratification algorithms to better understand which patients will do well in a model that includes remote visits and which will not.

Our work builds on others who have used remote physicians in acute care. In the traditional hospital setting, Gutierrez and colleagues^35^ demonstrated in a pre-post study the feasibility and acceptability of telehospitalist work for acutely ill adults on a medical ward but did not report on cases that could not be completed remotely. Kuperman and colleagues^36^ showed in a pre-post study that rural patients could receive safe care locally with a telehospitalist. Significant use of teleintensive care occurs, with likely a positive effect on mortality.^37,38,39^ In the home hospital setting, Sitammagari and colleagues^40^ demonstrated in a prospective case series a home hospital unit that used remote physician care for patients with COVID-19 with a reasonable safety profile (escalation rate, 13%; mortality, 0%). As noted above, our group was forced by the pandemic to deploy hybrid remote physician care for all patients, with good results.^41^ Compared with the current literature, this study adds a robust randomization schema and noninferiority analysis to examine the effect of remote physician care and allows for incorporation of remote care rather than strictly in-home care. Given the large number of new programs in the US, it also adds evidence for how to design in-home physician care.^42^ Many questions remain unanswered, including how deficits in remote care might be mitigated and where on the spectrum of remote vs in-home physician care is best.^43^

### Limitations

Our study has limitations. First, we were forced to cease enrollment early because of the COVID-19 pandemic, although we were still able to demonstrate noninferiority of our primary outcome and 2 of our 3 secondary outcomes (but chose not to adjust for multiple testing among our 3 secondary outcomes). Second, we enrolled at 2 sites, limiting generalizability, although patients’ demographic characteristics were diverse by race and ethnicity, payer, educational status, and others. Third, loss to follow-up could have led to nonresponse bias for the patient experience measures. Study staff made 3 telephone calls at different times to attempt to mitigate loss to follow-up. Fourth, our noninferiority limit for adverse events, although determined a priori, was sizable given the actual adverse event rate we found empirically. In-home physician care would likely have been superior in any tighter definition of noninferiority than that used in this study or if patients in the remote care group were not offered in-home physician care when remote care was deemed inadequate. Because of our noninferiority design, clinicians, program directors, and policy makers can interpret the upper bound of the CI with respect to any threshold they consider appropriate and ascertain whether it falls within the CI. The value of this study is to provide these data, not to set policy or dictate a model for a particular hospital. Fifth, we allowed for up to a month-long window to conduct patient experience follow-up surveys, which could have led to variable recall bias toward the null. Sixth, it was not possible to blind the research assistant ascertaining adverse event occurrence, although these outcomes are objective and well defined to minimize bias.

## Conclusions

When delivering acute care at home, remote physician care was generally acceptable and statistically noninferior to in-home physician care for patient safety and patient experience, although in-home physician visit capabilities when needed were critical to the remote physician group, and any system of remote physician care cannot be relied on in isolation. Given the home hospital model’s prior evidence base and newer data to suggest its role during COVID-19, expansion is important. Remote physician care may be a tool to significantly bolster a physician’s reach and efficiency when providing acute care, wherever a patient may be—at home, in the hospital, or elsewhere.^44,45^

## Notes

Supplement 1.
Trial Protocol

Supplement 2. **eAppendix**. Detailed Inclusion and Exclusion Criteria **eTable 1.** Characteristics of Patients Who Enrolled and Declined **eTable 2.** Characteristics of Patients Who Required In-home Visits vs Those Who Did Not, Among the Remote Group

Supplement 3.
Data Sharing Statement
