# Supplementary material for: Remote vs In-home Physician Visits for Hospital-Level Care at Home: A Randomized Clinical Trial
Source: JAMA Netw Open. 2022 Aug 30;5(8):e2229067. doi: 10.1001/jamanetworkopen.2022.29067 (PMC9428739; doi:10.1001/jamanetworkopen.2022.29067)
Supplement: Supplement 1. — Trial Protocol [file jamanetwopen-e2229067-s001.pdf]

Home Hospital for Acutely Ill Adults

DETAILED PROTOCOL
Version 4

BACKGROUND AND SIGNIFICANCE

Historical background

Hospitals are the standard of care for acute illness in the US, but hospital care is expensive and often unsafe, particularly for older individuals.[1] While admitted, 20% of older adults suffer delirium,[2] over 5% contract hospital-acquired infections,[3] and most lose functional status that is never regained.[4] Timely access to inpatient care is poor: many hospital wards are typically over 100% capacity, and emergency department waits can be protracted. Moreover, hospital care is increasingly costly: many internal medicine admissions have a negative margin (i.e., expenditures exceed hospital revenues) and incur patient debt.

The home hospital model and prior clinical studies

The home hospital model of care provides in-home acute care to acutely ill patients who would normally be admitted to a traditional hospital. This sort of substitutive care has been provided around the world for decades. Earlier studies of home hospital care in Australia,[5–8] Italy,[9] Spain,[10] and elsewhere demonstrated lower costs, while either maintaining or improving on quality, safety, and patient experience. In the United States, 2 principle studies of the home hospital model have demonstrated that a sizeable proportion of acute care can be delivered in the home with equal quality and safety, 20% reduced cost, and 20% improved patient experience.[11,12]

In late 2016, we launched a randomized controlled trial (RCT) of home hospital care, the first RCT to be performed in the US context. We first performed a small pilot with 21 patients. In short, we found the same as our international colleagues: cost savings while maintaining safety, quality, and patient experience.[13]

In July, 2017, we amended our protocol and relaunched the RCT at Brigham and Women's Hospital (BWH) and Brigham and Women's Faulkner Hospital (BWFH) with several key improvements. We added to our catchment zone (5-miles around both BWH and BWFH, not just BWH), removed certain exclusions (eg dementia), and added certain conditions (eg gout exacerbation). We subsequently enrolled 91 patients and published our findings, which corroborated prior work: lower cost, lower utilization, and improved physical activity. No large changes in quality or safety were observed, although the study was not powered to closely scrutinize these data.

Rationale

Our encouraging outcomes, coupled with the hospital's ongoing capacity concerns, led the hospital to require a single-arm intervention-only home hospital study for our current slate of included conditions that began in January, 2018. We have enrolled over 300 patients since January, 2018 very successfully and have consistently presented our safety data for the Committee's review. With our present local

data, compounded by the plethora of international studies cited above, we believe we have shown that home hospitalization delivers a reasonable substitute to hospitalization for correctly selected patients.

We seek to continue and expand this work in the context of IRB approval for several reasons, including the continued ability to

- employ devices in off-label uses. For example, the IRB previously approved the use of a wireless patch to record and report on vital signs. We continue to benefit from use of this device. We seek to deploy additional technologies to the home, all described below.
- deploy novel personnel. For example, the IRB previously approved the use of paramedics in our home hospital team. We intend to continue our use of paramedics, described below, and test in a retrospective pre/post design the impact of our already-deployed home hospital paramedic on safety and patient experience.
- Prospectively collect and report-on a battery of outcomes, many of which are first-in-class and will benefit from additional sample size. For example, the IRB previously approved collection of daily steps.
- Test remote physician care. In a randomized controlled non-inferiority trial, we will test an in-person daily physician visit ("in-person care;" control) vs a daily physician visit conducted remotely via video facilitated by a care team member ("remote care;" intervention) for patients who are home hospitalized. For patients in the remote care arm, the physician can pragmatically choose to see a patient in-person based on the clinical context.

Of importance, we have chosen to add 3 diagnoses (acute pancreatitis, inflammatory bowel disease exacerbation, and cystic fibrosis exacerbation) to this protocol that were originally planned to be randomized in our randomized controlled protocol (2016P001337). Due to volume constraints (high volumes of current diagnoses coupled with no budgeted ability to expand staff) we have not been able to start to care for these 3 new diagnoses but have carefully noted that we would be unlikely to enroll sufficient patients to make a RCT meaningful for these new diagnoses. We do now have the ability to hire additional staff members, allowing us to expand to these new diagnoses. We still believe that home hospital care could benefit these patients and want the opportunity to care for them. We will continue to closely vet each case for any safety issues and have any issues reviewed by our safety review committee as per our current protocol. With the IRB's approval, we will therefore move these diagnoses to the present protocol. We will continue to maintain protocol 2016P0001337 for bariatric surgery, as we are confident we can enroll a sufficient volume.

SPECIFIC AIMS

Primary Aim

We will accomplish a 20% reduction in hospitalization cost for selected hospitalized adults who would normally have been admitted to the hospital.

Secondary Aims

We will accomplish the following for the above-mentioned selected hospitalized adults:

- reduce health care utilization;
  - For example: decreased length of stay, number of labs/imaging, readmissions, ED visits
- maintain or improve on patient safety;
  - For example: reduced risk of delirium, falls
- maintain or improve on quality of care;
  - For example, high-value: increase in appropriate antibiotic selection, hours of sleep

    - For example, low-value: decrease in use of urinary catheter, inappropriate medications in the elderly
- maintain or improve on functional status and quality of life; and
    - For example: increase in EuroQol-5D-5L, activities of daily living scale
- improve absolute patient experience by 20%.
    - For example: increase in 3-item care transition measure, PROMIS experience measures

Please refer to our performance measures below for more details regarding the definitions and sources for each of these measures.

SUBJECT SELECTION

Inclusion/exclusion criteria

Inclusion

- Social
    - Resides within either a 7.5-mile driving radius of the BWFH emergency room
        - Driving radius will be obtained via inputting a nearby (but not exact) address into Google Maps.
    - Has capacity to consent to study OR can assent to study and has proxy who can consent (see subject enrollment, below)
    - Can identify a potential caregiver who agrees to stay with patient for first 24 hours of admission. Caregiver must be competent to call care team if a problem is evident to her/him. After 24 hours, this caregiver should be available for as-needed spot checks on the patient.
        - This criterion may be waived for highly competent patients at the patient and clinician's discretion.
- Clinical
    - >=18 years old
    - Primary or possible diagnosis of cellulitis, heart failure, complicated urinary tract infection, pneumonia, COPD/asthma, other infection, chronic kidney disease, malignant pain, diabetes and its complications, gout flare, hypertensive urgency, previously diagnosed atrial fibrillation with rapid ventricular response, anticoagulation needs, or a patient who desires only medical management that requires inpatient admission, as determined by the emergency room team.
        - Regarding anticoagulation needs, this includes a patient who requires therapeutic anticoagulation and concomitant monitoring (thus requiring inpatient status)
        - Regarding a patient who desires only medical management, this includes a patient who requires acute care for symptom management, but declines any surgical intervention. This may include a patient who is about to transition to hospice care, for example, but still has the functional capacity to meet our criteria below. Under these circumstances, we would make sure that various contingencies, including possible transition to hospice care or hospital readmission, are completely understood by patients and caregivers as applicable.
        - In order to achieve enrollment goals, we *a priori* reserve the ability to expand this criterion to include patients who fail observation status and patients who

are transferred to the observation unit under the care of the emergency room observation team.
    - Regarding IBD
        - Requires intravenous infusion and acute care monitoring per outpatient gastroenterologist
    - Regarding acute pancreatitis
        - Please refer to attachment
    - Regarding exacerbation of cystic fibrosis or non-cystic fibrosis bronchiectasis
        - Exacerbation (>=4/12)
            - Change in sputum
            - New or increased hemoptysis
            - Increased cough
            - Increased dyspnea
            - Malaise, fatigue of lethargy
            - Temperature above 38°C
            - Anorexia or weight loss
            - Sinus pain or tenderness
            - Change in sinus discharge
            - Change in physical examination of the chest;
            - Decrease in pulmonary function by 10 percent or more from a previously recorded value
            - Radiographic changes indicative of pulmonary infection

- Location of presentation
    - Emergency department
        - Decision to admit or observe patient made by emergency department
    - Hospital floor
        - Patient is clinically stable with a clear plan for continued acute care, as agreed upon by the inpatient and home hospital teams
        - All inclusion and exclusion criteria must still be fulfilled
    - Home
        - Patient is known to the home hospital team from a prior home hospitalization
        - All inclusion and exclusion criteria must still be fulfilled. This requires the same clinically warranted workup as the patient would have received in the emergency department or upon arrival to the floor, including imaging and other diagnostics. Should these be warranted and not be available (e.g., expedited CT imaging), the patient would be excluded.
    - Clinic
        - Patient is well-known by a referring clinician who is confident of the diagnosis and management after a history, physical, and if warranted diagnostic testing in the clinic. For example, a cardiologist might refer a patient with known heart failure from clinic who has volume overload and would otherwise directly admit her/his patient to the floor.
        - All inclusion and exclusion criteria must still be fulfilled. This requires the same clinically warranted workup as the patient would have received in the emergency department or upon arrival to the floor, including imaging and other diagnostics. Should these be warranted and not be available (e.g., expedited formal TTE), the patient would be excluded.

Exclusion

- Social
    - Undomiciled
    - No working heat (October-April), no working air conditioning if forecast > 80°F (June-September), or no running water
    - On methadone requiring daily pickup of medication
    - In police custody
    - Resides in facility that provides on-site medical care (e.g., skilled nursing facility)
    - Domestic violence screen positive[14]
- Clinical
    - Acute delirium, as determined by the Confusion Assessment Method[2]
    - Cannot establish peripheral access in emergency department (or access requires ultrasound guidance, unless point-of-care ultrasound is available)
    - Secondary condition: end-stage renal disease on hemodialysis, acute myocardial infarction, acute cerebral vascular accident, acute hemorrhage
    - Primary diagnosis requires multiple or routine administrations of intravenous narcotics for pain control
    - Cannot independently ambulate to bedside commode, unless home-based aides are available
    - As deemed by on-call MD, patient likely to require any of the following procedures: computed tomography, magnetic resonance imaging, endoscopic procedure, blood transfusion, cardiac stress test, or surgery (unless expedited pathway for these procedures is available)
    - For pneumonia:
        - Most recent CURB65 > 3: new confusion, BUN > 19mg/dL, respiratory rate>=30/min, systolic blood pressure<90mmHg, Age>=65 (<14% 30-day mortality)[15]
        - Most recent SMRTCO > 2: systolic blood pressure < 90mmHg (2pts), multilobar CXR involvement (1pt), respiratory rate >= 30/min, heart rate >= 125, new confusion, oxygen saturation <= 90% (<10% chance of intensive respiratory or vasopressor support)[16]
        - Absence of clear infiltrate on imaging
        - Cavitary lesion on imaging
        - Pulmonary effusion of unknown etiology
        - O_2 saturation < 90% despite 5L O_2
    - For heart failure:
        - Has a left ventricular assist device
        - GWTG-HF[17] (>10% in-hospital mortality) or ADHERE[18] (high risk or intermediate risk 1)*
        - Severe pulmonary hypertension
    - For complicated urinary tract infection:
        - Absence of pyuria
        - Most recent qSOFA > 1 (SBP≤100 mmHg, RR≥22, GCS<15 [any AMS]) (if sepsis, >10% mortality)[19]
    - For other infection
        - Most recent qSOFA > 1 (SBP≤100 mmHg, RR≥22, GCS<15 [any AMS]) (if sepsis, >10% mortality)[19]
    - For COPD

        - BAP-65 score > 3 (BUN>25, altered mental status, HR>109, age>65) (<13% chance in-hospital mortality): exercise caution
    - For asthma
        - Peak expiratory flow < 50% of normal: exercise caution
    - For diabetes and its complications
        - Requires IV insulin
    - For hypertensive urgency
        - Systolic blood pressure > 190 mmHg
        - Evidence of end-organ damage; for example, acute kidney injury, focal neurologic deficits, myocardial infarction
    - For atrial fibrillation with rapid ventricular response
        - Likely to require cardioversion
        - New atrial fibrillation with rapid ventricular response
        - Unstable blood pressure, respiratory rate, or oxygenation
        - Despite IV beta and/or calcium channel blockade in the emergency department, HR remains > 125 and SBP remains different than baseline
        - Less than 1 hour of time has elapsed with HR < 125 and SBP similar or higher than baseline
    - For patients with end-stage renal disease on peritoneal dialysis
        - Peritoneal catheter malfunction
        - Requires temporary hemodialysis
    - For exacerbation of IBD
        - Fever > 102F
        - Rigid/surgical abdomen
        - Acute intestinal obstruction, perforation, or toxic megacolon
        - History of intestinal obstruction, perforation, toxic megacolon
        - Abscess or abdominal mass
        - Likely to require total parenteral nutrition
        - Likely to require blood transfusion
    - For acute pancreatitis
        - Please refer to attachment
    - For exacerbation of cystic fibrosis or non-cystic fibrosis bronchiectasis
        - Pneumonia criteria above
        - Respiratory failure
        - Moderate or large-volume hemoptysis (>=10mL)
        - Pneumothorax
        - Pancreatitis
        - Post-transplant
- Home hospital census is full (maximum 16 patients at any time)

*GWTG-HF: AHA Get with the Guidelines: SBP, BUN, Na, Age, HR, Black race, COPD
ADHERE: Acute decompensated heart failure national registry: BUN, creatinine, SBP

Source of subjects and recruitment methods

Subjects are eligible if presenting from 4 settings, as outlined above (“location of presentation”):

- Emergency departments at BWH and BWFH
- Inpatient wards at BWH and BWFH

- Patient homes of previously home hospitalized patients
- BWH and BWFH clinics
- Those meeting primary diagnosis, age, and geographic requirements will be screened by our research assistants for further enrollment (below).

Subject Enrollment

Methods of enrollment, Procedures for obtaining consent

- Decision to admit or observe made by emergency room team or by an ambulatory clinician who would otherwise admit her/his patient.
- Participant is pre-screened by a research assistant for primary diagnosis, age, and residence within 7.5 miles via the electronic medical record.
- If participant meets those criteria, the emergency room team or ambulatory clinician will ask the patient/caregiver if the research assistant can approach the patient regarding the home hospital study.
- If participant agrees, the research assistant will describe the program, answer initial questions, and further assess whether the participant meets inclusion/exclusion non-clinical (e.g., social) criteria. The RA will then contact the on-call MD to assess clinical criteria by EHR review.
- If the participant has interest in the program and meets inclusion/exclusion criteria, the on-call MD (either in-person or via a live video visit facilitated by the research assistant) will obtain written informed consent of the participant to enroll in the home hospital program.
- For a subject known to be without capacity, any of the following individuals (listed in general order of preference) may give consent provided they are on-site and able to sign the informed consent (the patient will assent):
    - court appointed guardian with specific authority to consent to participation in research or authority to make health care decisions for a class of diagnostic and therapeutic decisions inclusive of the proposed research;
    - health care proxy/person with durable power of attorney with specific authority for making health care decisions inclusive of the proposed research; or
    - spouse, adult child, or other close family member who knows the subject well and has been involved in their care.
    - When surrogate consent is relied upon, the Investigator must ensure that the surrogate understands that his or her decisions should be based on "substituted judgment." This means that the decision reflects a potential subject's own views when s/he had the capacity to express them. If a potential subject did not previously express a view on the matter, the surrogate should make the decision based on the potential subject's best interests.
    - When surrogate consent is relied upon, assent of the subject will be a requirement for participation in the research unless the subject is incapable of giving assent due to his/her medical condition. If the individual objects to participation, s/he will not be enrolled.
    - In the case of surrogate consent, a patient must be returning to their prior level of supervision.
- For patients already on the hospital floor: Previously, we had only allowed patients who had been admitted for less than 24 hours to be home hospitalized. We now wish to expand this criterion to patients at any timepoint in their hospitalization. We believe that many patients may benefit from home hospital care, irrespective of how long they have been traditionally

hospitalized. Enrollment will proceed exactly as in the emergency department, with the exception that it will need to be assessed and reassessed daily with an inpatient team to determine the time at which the patient has a stable acute care plan that meets all of the above criteria. That is, the patient is clinically improving, has continued low probability of clinical deterioration or need for an invasive procedure, but still requires acute care.
- Subjects who do not speak English will use a short-form consent form with the assistance of a certified telephone interpreter.

Treatment Assignment

- All patients will be offered home hospitalization.
- If patient/caregiver does not consent, the research assistant will ask the patient's permission to collect covariates, functional status, and a quality of life measure to determine generalizability of enrollment.

As an amendment to our current protocol, patients will be randomized (random sized blocks between 3 and 6 stratified by infection, heart failure, COPD/asthma, and other with concealed allocation) to remote care (intervention) versus in-person care (control) at the time of enrollment. Once 210 patients have been enrolled in this schema (see power calculations below), and if remote care is noninferior to in-person care, we will allow remote care to continue as a single arm while continuing other concurrent experiments.

STUDY PROCEDURES

Study visits and measured parameters

Each day, the home hospital team will make visits to the patient's home. These are personnel who have been trained in home health and carry out home health as per the usual care they deliver. The home hospital module offers most of the same medical components that are standard of care in an acute care hospital (**Figure below**). The typical staff (MD, RN, paramedic, case manager), diagnostics (blood tests, vital signs, telemetry, x-ray, and ultrasound), intravenous therapy, and oxygen/nebulizer therapy will all be available for home hospital. Optional deployment of food, home health aide, physical therapist, occupational therapist, community health worker, and social worker will be tailored to patient need. An enrolled subject will have a conversation with study staff regarding which staff will visit their home. These staff may change as a patient's needs are further assessed throughout the admission. Home hospital improves upon the components of a typical ward's standard of care in several ways:

- Point of care blood diagnostics (results at the bedside in <5 minutes);
- Minimally invasive continuous vital signs, telemetry, and activity tracking, including clinical deterioration algorithms, steps taken, and sleep quality;
- 24/7 clinician video visits;
- 12 to 1 patient to MD ratio, compared to typical 16 to 1;
- Ambulatory/portable infusion pumps that can be worn on the hip;
- Optional access to a personal home health aide and community health worker

Component	Module
Personnel	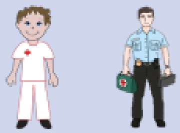 **Nurse/Paramedic**<br>• Minimum 2 daily visits<br>• Medication administration<br>• IV access management<br>• Education<br>+<br>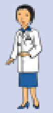 **Physician**<br>• 1 daily interaction<br>• Medical decision making<br>±<br>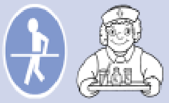 **Home Health Team**<br>• Home health aide<br>• Home PT/OT<br>• Social Worker<br>• CHW
Diagnostics	Point of care: BMP, Hgb/Hct, PT/INR, ultrasound, x-ray, PFTs, ECG
Pharmacy	Any inpatient medication, most any route
Equipment	Ambulatory infusion pump, peripheral IV, oxygen concentrator
Communication	Telepresence/virtual visits
Monitoring	Wireless remote vital signs, telemetry, activity tracking, sleep tracking, predictive analytics for deterioration

As denoted in the figure above, the physician will make at least one daily interaction with the patient. The nature of this interaction will be guided by the control versus intervention arm, and the physician's discretion. If the patient is in the control arm, the physician will make a daily in-person visit (usual care). If the patient is in the intervention arm, the physician sees the patient remotely via facilitated video, including near-real-time transmission of heart, lung, and abdominal sounds as needed. The nurse or paramedic seeing the patient will facilitate a physical exam and discussion with the patient. If the physician chooses for clinical purposes, s/he may still see the patient in-person. At the discretion of the physician, paramedic, and nurse, the physician can make additional visits should the patient's clinical condition require it. The nurse or paramedic will make at least two daily visits to the patient's home. At the discretion of the physician, paramedic, and nurse, the nurse or paramedic can make additional visits should the patient's clinical condition require it. This will be an ongoing twice daily discussion among clinicians, patient, and caregivers, as applicable.

As denoted in the figure above, a patient or clinician can initiate a video or in-person visit at any time.

As denoted in the figure above, a home health aide may be deployed. If deemed necessary by the home hospital attending, patient, and/or caregivers, the home hospital program can provide an on-demand home health aide that can provide care in 12 hour shifts.

As denoted in the figure above, the home hospital team uses a suite of communication software to communicate with the patient and among team members. The home hospital team has the option of using a home hospital dashboard that combines a patient list with communication (video, text) and visualization of data from monitoring devices (VitalPatch, for example). Clinician-facing tools: patient list, communication (video, text, audio), and visualization of and alerting on data from monitoring devices (VitalPatch, for example). Patient-facing tools: plan for the day, calendar for the day, reminder

from the care team, a simple snapshot of sleep and activity, and the ability to contact the care team via video, text, or audio . Caregiver-facing tools: same as patient-facing tools, if permission is granted by the patient or health care proxy.

For patients with chronic/stable dementia, they will receive mental status assessments (eg, Confusion Assessment Method) by the home hospital RN or paramedic at each visit (at least 2 daily). As is standard of care, we will take measures to avoid delirium: open blinds to allow light in during the day, minimize environmental noise, encourage typical routines to optimize eating and sleeping, and reassess medication list for culprit medications.

Clinical parameters measured will be at the discretion of the physician, paramedic, and nurse, who treat the participant following evidence-based practice guidelines, just as in the usual care setting. Monitoring and data collection will be performed by home hospital clinicians. The clinical home hospital team manually checks vital signs at least twice daily. If deemed necessary based on a patient's acuity, the home hospital team can visit additional times during the day and a dedicated overnight aide can be deployed to check vital signs multiple times throughout the night. At all times, the patient and care team have 24/7 access to the home hospital attending physician, who can be in the patient's home in less than 20 minutes at any time of day. On top of this "on-the-ground" team, the home hospital attending is notified with an alarm should there be delayed data transfer. Such an alarm would cause the attending to contact the patient as deemed clinically appropriate. Please see below for all collected data points.

Documentation by RN, paramedic, and MD will occur via the hospital's electronic health record. Clinical photos and flowsheets that cannot be stored in the EHR will be compiled in a Partners encrypted share drive.

Additional personnel descriptions from those listed in the Figure:

- Community health worker (CHW): a trained and certified lay person who often shares culture and/or community with the patient. The CHW strives to improve familiarity with the plan of care, improve adherence to the plan of care (medications, appointments), improve clinical outcomes, and help the patient manage social barriers to care (e.g., applying for medication financial assistance). The CHW will make once daily visits during the admission and twice weekly visits after discharge for 30 days. The CHW will have no role in providing medical decision-making or in nursing care.
- Community paramedic: a paramedic will provide in-home clinical care under the supervision of the home hospital attending. The paramedic can give medications under the direction of the attending. This paramedic will receive additional training from a collaborative group of emergency and internal medicine physicians at Brigham who have experience in community paramedicine. Our experience to-date with our paramedic has been highly beneficial to our team and patients. The paramedic will receive quality-assurance as per standard EMS guidelines.
- Medical resident: a BWH PGY2 or PGY3 in internal medicine will have the option to select home hospital service as a two-week elective. They will attend a home hospital training and continue to have twice-weekly home-based medicine seminars throughout. Their role includes daily rounds for supervised medical decision-making, responses to patient needs, and documentation. They will always have to travel with another team member when visiting a

patient's home. The home hospital attending will supervise the resident through direct observation and making addendums to documentation.

Prospective Data Collection for artificial intelligence algorithms

- An additional RA (separate from the RA who enrolls patients in the home hospital study) will visit patient homes following their morning clinical visit to obtain additional gold-standard data needed to train several artificial intelligence algorithms. All questionnaires and samples obtained are included in the supplement.

As part of a small feasibility pilot (5 subjects) in conjunction with Mount Sinai Health System, we will test the feasibility of obtaining a short battery of questions related to "post-hospital syndrome," with the ultimate goal of ascertaining if home hospital care reduces post-hospital syndrome. Both Mount Sinai and Brigham are collecting these data as part of a pilot (see attached and below, "Post-Hospital Syndrome measures.") We will share with Mount Sinai the results of the questionnaire, age, gender, education, race/ethnicity, admitting diagnosis, blood pressure, heart rate, all-cause hospital readmissions, stand-alone emergency department visits, transfers to a skilled nursing facility, and referral to certified home health agency.

Drugs to be used

Only medications used in the usual care of hospitalized patients will be used. For example, a patient with pneumonia might receive guideline-based antibiotics with ceftriaxone and azithromycin.

Controlled substances:

- In order to care for several conditions, we may require controlled substances. We have, through expert consensus groups, developed protocols that minimize or entirely reduce controlled substance requirements.
- Controlled substances are commonly used in the home, whether for malignant or chronic pain, end of life, and other circumstances.
- Together with the Brigham pharmacy, Office of the General Counsel, and our home hospital clinicians (MDs, paramedics, and RNs), we have carefully created a controlled substances use policy that allows us to use controlled substances while protecting our clinicians and our patients and their caregivers.
- Please see this new policy as a separate attachment.

Devices to be used

- Home hospital employs standard devices during the usual care of hospitalized patients. For example, a nebulizer machine.
- Home hospital additionally employs
    - Vital signs monitoring patch: Vital Connect VitalPatch (FDA cleared application/use, but new hardware and software updates are pending clearance)
        - As we have done in the past under IRB approval, we will use new Vital Connect VitalPatch hardware and VistaCenter software as they become available. The newest hardware, the VitalPatch 2.0, and newest software, VistaCenter 2.0 add core temperature, longer battery life, and connectivity for a blood pressure cuff and pulse oximeter. Importantly, these only represent new functionality, but

these versions are not FDA approved. There will be no decrement in our ability to monitor patients.

- Ambulatory infusion pump: Smiths Medical CADD Solis VIP (FDA cleared application/use)
- Point of care diagnostic meter: Abbott iSTAT (FDA cleared application/use) or Siemens Epoc (FDA cleared application/use)
- Ambulatory ECG: General Electric ECG (FDA cleared application/use)
- Massimo Rad-7 Rainbow Acoustic respiratory monitor (FDA cleared application/use)
- Digital stethoscope: Eko (FDA cleared application/use)
    - Our home hospital team plans to use this FDA cleared digital stethoscope to transmit near-real-time heart, lung, and abdominal sounds to a remote physician.
- Point of care ultrasound (POCUS)
    - Our home hospital team wants to improve the timeliness and efficiency of our radiology studies. The best way to do this is to add POCUS capabilities for our clinicians.
    - We will train our clinicians per standard Brigham protocols for POCUS, as approved by Dale Adler. They will have standard oversight and overreading.
    - Point of care ultrasound device
        - Phillips Lumify Ultrasound (FDA cleared application/use)
            - We may use this device to assist clinicians with diagnosis and treatment. Examples include
                - insertion of a peripheral intravenous catheter;
                - assessment of heart and lung function; and
                - assessment of post-void residual
            - This system is already in use at Brigham.
- Continuous glucose monitor: Dexcom G6 (FDA cleared application/use at home, not in the acute setting with off-label cleaning procedure)
    - Continuous glucose monitoring in the acute setting has been shown to be safe and accurate, although further testing is ongoing.[20–22]
    - To improve our ability to monitor and care for patients who have glycemia concerns, we will begin using the Dexcom G6. This will provide our clinicians continuous glucose measurements and predictive glucose alerts (signaling hypoglycemia or hyperglycemia), while reducing finger stick glucose checks.
    - Any patient who the home hospital physician believes would benefit from continuous glucose monitoring, will receive the Dexcom G6 during their home hospital stay.
    - Although the Dexcom G6 is FDA cleared, the transmitter's cleaning procedure is not. The Dexcom G4 (a highly similar unit) does have an FDA approved cleaning procedure. We propose (upon recommendation of Dexcom) to use this cleaning procedure with the Dexcom G6 (see procedure attached as separate document). The sensor that inserts into the patient is single-use only.
    - This device will never interact with Brigham computer systems, never be programmed with protected health information, and never be used in the hospital.
- Blood alcohol content breathalyzer: BACtrack S80 Pro (FDA cleared application/use)

        - We would like the ability to perform point of care testing for blood alcohol content.
        - We plan to use the FDA-cleared BACtrack S80 Pro, manufactured by BACtrack.
        - This is a free-standing device that has no send/receive capability, will never interact with Brigham computer systems, never be programmed with protected health information, and never be used in the hospital.
    - Virtual physical therapy
        - Prior literature has shown that movement is paramount to healing and maintaining health. We would like the ability to offer more intensive physical therapy to our home hospital patients with the use of a virtual physical therapy system called VeraHome, created by Reflexion Health.
        - VeraHome is a virtual physical therapist that is a combination of video and avatar-based interactions. The first interaction is by video with an actual physical therapist. The therapist evaluates the patient and prescribes an exercise program. The patient, with the as-needed assistance of the home hospital team and/or caregivers, subsequently performs the exercise program led by the Vera avatar. The avatar is able to monitor the patient's movements with the included depth camera, and able to provide real-time feedback regarding adequate movement. These details are fed back to the physical therapist, who as needed and at least once weekly, meets with the patient by video and makes adjustments to the exercise program. If the patient is not progressing well with virtual physical therapy, the option always exists to use traditional physical therapy.
        - VeraHome is FDA cleared for upper extremity physical therapy. It is awaiting approval for lower extremity physical therapy. A possible risk of using this system is harming oneself during physical therapy exercises. Given our at least twice daily visits to patients' homes and continuous biometric monitoring, we foresee this to be a minimal risk.
        - Reflexion has completed its Cybersecurity assessment.
    - Predictive analytics for deterioration and improvement
        - We want to optimize the use of biometric data we collect from the VitalPatch. Simply examining heart rate or respiratory rate alone to predict health improvement or deterioration is likely less powerful than a combined set of data points (for example: heart rate + respiration rate + ECG + motion + stress + Sleep analysis + temperature). Also, methodically examining hour-by-hour changes in these vitals could improve clinical recognition of impending problems.
        - We have partnered with Biofourmis, a predictive analytics company with expertise in analyzing multiple biosensor output for impending clinical changes. Biofourmis' AI platform, biovitals™ uses advanced machine learning and physiology science resulting in accurate detection of patient's health deterioration or improvement. Their technology is currently in use at the Mayo Clinic to predict exacerbations of heart failure based on multiple biosensor data including ECG + Heart rate + respiration rate + activity + accelerometer data.
        - We will add their current analytics engine to our current alarm systems, as well as co-develop new state-of-the-art analytics. Our data will be processed in their HIPAA-compliant cloud and alarms sent back to our clinicians.

      - Biofourmis has completed their cybersecurity assessment and has been vetted by the Partners data sharing committee.
      - Their current analytics engine includes detection of the following:
        - Automated, real-time analysis of ECG data to detect cardiac arrhythmias.
        - Multivariate analysis of multiple physiology data using biovitals™ analytics engine to accurately detect (including alarms) health deterioration or health improvement – which is quantified as the biovitals Index™. The analytics engine, biovitals™, dynamically learns the patient's physiology and context (including activity intensity, posture, position, steps, etc.) to dynamically build a personalized signature and is able to compute subtle physiology changes which are indicators of the patient's health deterioration.
        - Processing of raw accelerometry to derive activity intensity, steps, position, posture, rotation etc.
        - Processing of raw accelerometer and other physiology data for sleep analysis, including sleep duration, sleep latency, sleep stages etc.
        - Processing of ECG data (specifically using Inter-beat-Interval or R-R interval) to detect cardiac arrhythmias, using Poincare plots.
      - We plan 3 stages
        - First, we will perform a retrospective analysis on existing patient data. We will annotate existing patient data through chart review and clinician interviews, then test the performance of biovitals™, as well as newly created alarms, to gold-standard annotators.
        - Second, we will collect prospective data to enhance annotations and teach novel algorithms. This additional step was recognized as necessary in order to provide gold-standard data for desired algorithms.
        - Third, we will add biovitals™, as well as newly created alarms, to our current vital-sign based alarms, such that there is no possibility of a decrement, but only an improvement, in safety.
    - Home hospital portable medical bed
      - Beds in patient's home are often lacking in their ability to mobilize the patient, yet "hospital-level" beds are usually too expensive or unavailable to patients in their home. We have partnered with HillRom to codevelop a portable bed insert that can provide many of the features of a hospital bed. This device is inserted underneath a standard mattress and can elevate the head and foot of bed.
      - While extremely unlikely, should the bed malfunction (deflate), it possible that a patient could lose elevation/incline while sleeping, which could be distressing to the patient.

Procedures/surgical interventions

- Home hospital employs the same standard procedures used during the usual care of hospitalized patients. For example, peripheral venipuncture. Need for surgery is an exclusion criterion.

Data to be collected and when the data is to be collected

Cost

Measure	Source(s)	Day(s) Obtained
Total cost, acute care episode	Home hospital accounting	Discharge
Total cost, 30-days post discharge	Partners data	+30
Total cost, acute care episode plus 30-days	As above	+30

Health Care Utilization

Measure	Source	Day(s) Obtained
Length of stay, days	RA via MD/RN for home patients; RA EHR review for inpatients	Discharge
IV medication, days	As above.	Daily
Intravenous fluids, days	RA via EHR	Daily
Intravenous diuretics, days	RA via EHR	Daily
Intravenous antibiotics, days	RA via EHR	Daily
Oxygen requirement, days	RA via RN	Daily
Nebulizer treatment, days	RA via EHR	Daily
Imaging, #	As above. +30 will be cross-checked with Partners data.	Daily, +30
Lab Orders, #	As above.	Daily, +30
MD sessions, # of notes	As above.	Daily, +30
Consultant sessions, # of notes	As above.	Daily, +30
PT/OT sessions, # of notes	As above.	Daily, +30
Disposition (routine, SNF, home health, other)	RA via MD/RN for home patients; RA EHR review for inpatients	Discharge
PCP follow-up within 14 days, y/n	RA via pt phone call, cross-checked with Partners data.	+30
SNF utilization, days	As above.	+30
Home health utilization, days	As above.	+30
All-cause readmission(s) after index, # and y/n + date	As above.	+30
Unplanned readmission(s) after index, # and y/n + date	As above.	+30
ED observation stay(s), # and y/n + date	As above.	+30
ED visit(s), # and y/n + date	As above.	+30

RA: Research assistant

Safety

Measure	Source	Day(s) Obtained
Fall	RA via MD/RN for home patients; MD EHR review for inpatients	Daily

Delirium, y/n and time to resolution	RNs in home and inpatient settings calculate the CAM (standard of care in both). RA will obtain the home patient CAM via the home RN; RA will obtain the inpatient CAM via EHR.	Daily
Potentially preventable VTE	RA via MD/RN for home patients; MD EHR review for inpatients	Daily
New pressure ulcer	As above.	Daily
Thrombophlebitis at peripheral IV site	As above.	Daily
Hospital Acquired Condition		
CAUTI	As above.	Daily
Clostridium difficile	As above.	Daily
MRSA	As above.	Daily
Transfer back to hospital[a]	RA via MD/RN	Discharge
All-cause mortality during admission	RA via MD/RN for home patients; MD EHR review for inpatients	Discharge
Unplanned mortality during admission	RA via MD/RN for home patients; MD EHR review for inpatients	Discharge
All-cause 30-day mortality	RA via pt/caregiver call	+30
Unplanned 30-day mortality	RA via pt/caregiver call	+30
Heart Failure		
New arrhythmia	RA via MD/RN for home patients; MD EHR review for inpatients	Daily
Hypokalemia	As above.	Daily
Acute kidney injury	As above.	Daily

[a]: intervention arm only

Quality: High-Value Care

Measure	Source	Day(s) Obtained
Pain management	RA via MD/RN for home patients; RA EHR review for inpatients	Daily
Hours of sleep per day	RA via activity tracker	Daily
Hours of sleep per night	RA via activity tracker	Daily
Hours of activity per day	RA via activity tracker	Daily
Hours of activity per night	RA via activity tracker	Daily
Hours of sitting upright per day	RA via activity tracker	Daily
Hours of sitting upright per night	RA via activity tracker	Daily
Daily steps	RA via activity tracker	Daily
Pneumonia		
Pneumococcal vaccination if appropriate	RA via EHR	Discharge
Influenza vaccination if appropriate	RA via EHR	Discharge

Smoking cessation counseling if appropriate	RA via EHR	Discharge
Heart Failure		
Evaluation of ejection fraction scheduled or completed if not done within 1 year	RA via EHR	Discharge
ACEI/ARB for HFrEF (EF < 40%)	RA via EHR, pt (post discharge)	Discharge, +30
Beta blocker for HFrEF (EF < 40%)	As above.	Discharge, +30
Aldosterone antagonist for HFrEF (EF < 40%)	As above.	Discharge, +30
Lipid lowering for CAD, PVD, CVA, or diabetes	As above.	Discharge, +30
Smoking cessation counseling if appropriate	As above.	Discharge
Smoking status post-discharge	RA via pt	+30
Glucose		
Patient-days with glucose between 70mg/dL and 180mg/dL	Dexcom device and EHR	Daily
Patient-days with glucose > 300mg/dL	Dexcom device and EHR	Daily
Patient-days with glucose < 70mg/dL	Dexcom device and EHR	Daily
Patient-days with glucose < 40mg/dL	Dexcom device and EHR	Daily
Patient-day weighted mean glucose	Average all readings for one day, then average the averages across all days	Daily
Any glucose >300mg/dL, y/n	Dexcom device and EHR	Daily
Any glucose <70mg/dL, y/n	Dexcom device and EHR	Daily
Any glucose <40mg/dL, y/n	Dexcom device and EHR	Daily
Capillary versus continuous glucose (see statistical methods)	Dexcom device and EHR	Daily

Quality: Low-Value Care

Measure	Source	Day(s) Obtained
Use of inappropriate medications in the elderly	RA via EHR/MAR	Daily
Use of foley catheter	RA via MD/RN for home patients; EHR review for inpatients	Daily
Use of restraints	RA via MD/RN for home patients; EHR review for inpatients	Daily

Patient Functional Status and Quality of Life

Measure	Source	Day(s) Obtained
EuroQol -5D-5L	RA via patient	Admission, discharge, +30
SF-1	RA via patient	-30, admission, discharge, +30

Activities of daily living: admission to discharge; admission to 30-day post-discharge	RA via patient	-30, admission, discharge, +30
Instrumental activities of daily living: admission to discharge; admission to 30-day post-discharge	RA via patient	-30, admission, discharge, +30
Patient Health Questionnaire-2	RA via patient	Admission, discharge, +30
PROMIS Emotional Support Short Form 4a	RA via patient	Admission, discharge, +30
Days at home since discharge	RA via patient	+30
Milestones		
Walk around ward/home	RA via RN	Discharge
Get to (non-commode) bathroom	RA via RN	Discharge
Walk 1 flight of stairs	RA via RN	Discharge
Visit with friends/family	RA via patient	Discharge
Walk outside around my home	RA via patient	+30
Go shopping	RA via patient	+30
Post-Hospital Syndrome measures	RA via patient	Admission, +3, +30

Patient and Family Experience

Measure	Source	Day(s) Obtained
3-item Care Transition Measure	RA via patient	+30
Picker Experience Questionnaire	RA via patient	+30
Global satisfaction	RA via patient	+30
Qualitative interviews	See Below	+30

Process Measures

Measure	Source	Day(s) Obtained
Time from admission decision to assessment by RA	RA	Admission
Time from RA assessment to dismissal	RA	Admission
Time from arrival home or to floor and MD evaluation	RA via MD	Admission
Time from arrival home or to floor and RN evaluation	RA via RN	Admission
RN to patient ratio	RA via census	Daily
Number of RN visits	RA via RN/EHR	Daily
Number of "on call" MD interactions (video or phone)	RA via MD	Daily
Number of "on call" MD in-person visits	RA via MD	Daily
Duration of 1st RN visit	RA via RN	Daily
Duration of subsequent RN visit	RA via RN	Daily
Clinician focus group	All clinicians	Post-study
Failed stethoscope transmissions, number and %	RA via clinicians	Daily

Artificial Intelligence Measures

Measure	Source	Day(s) Obtained
Specificity		
Sensitivity		
Positive predictive value		
Negative predictive value		
Accuracy of categorization by emergent, significant, and borderline, and normal (as outlined by Smith et al)[23]		
ECGs with no undercalls and no overcalls, stratified by all, with >=1 abnormality, >=1 major abnormality, >=1 emergency (as outlined by Smith et al)		
Timed Walking	RA	Daily
Timed Stairs	RA	Daily
Gait	RA	Daily
Use of Assistive Device	RA	Daily
Balance Questions to assess Fall risk	Patient	Daily
Timed Up and Go Balance Test	RA	Daily
30 Second Chair Stand Test	RA	Daily
4 Stage Balance Test	RA	Daily
Loss of Consciousness in the past 24 hours	Patient	Daily
Cough and Inducible Cough	RA	As needed
Respiratory Rate	RA	3-6x Daily
Inspiratory to Expiratory Ratio	RA	1-2x Daily
Orthostatic Vitals	RA	1-2x Daily
Lab Values via capillary stick (used only for research)	RA	1-2x Daily
Acoustic Respiratory Rate	RA	Daily
Number of times patient got up during the night (to urinate)	Patient	Daily
Richards-Campbell Sleep Questionnaire	Patient	Daily
STOP-Bang Questionnaire	Patient	On admission
10 Question Perceived Stress Questionnaire	Patient	Daily
Live Telemetry Alarms	Doctor, Nurse, EMT	As they occur

For electrocardiographic categorizations, see Smith et al
For nonelectrocardiographic categorizations, see Appendix

Covariates of Interest

Measure	Source	Day(s) Obtained
Age	RA via EHR[a]	Admission
Gender	RA via EHR	Admission
Race/ethnicity	RA via patient	Admission
Primary language	RA via EHR	Admission
Health insurance status, public/private/none	RA via EHR	Admission
BMI	RA via EHR	Admission

Comorbidities, type and #	RA via EHR and H&P	Admission
Partner status	RA via patient	Admission
Education	RA via patient	Admission
Zip code	RA via EHR	Admission
Employment	RA via H&P	Admission
Smoking status	RA via H&P	Admission
Medications used as outpatient, #	RA via EHR	Admission
DNR/I code status	RA via H&P	Admission
Home health aide prior to admission	RA via patient	Admission
Readmission risk score on discharge (HOSPITAL)	RA via EHR	Discharge
Elective and urgent admissions in the previous year, #	RA via EHR and patient	Admission
ED visits in the previous 6 months, #	RA via EHR and patient	Admission
Interqual disease-specific leveling	RN	Admission
PRISMA-7	RA via patient	Admission
Eight-item Interview to Differentiate Aging and Dementia	RA via patient or proxy	Admission
Would you be surprised if this patient died in the next year?	RA via MD	Admission
BRIEF health literacy screening tool	RA via patient	Admission
Caregiver burden	RA via caregiver	Admission, +30
Lives alone	RA via patient	Admission

[a]: Note that RA will recheck covariates after MD H&P completed to ensure comorbidities and other covariates correctly captured

Standard Operating Procedure for Problematic Situations

Any subjects presenting with problematic situations will be immediately transferred back to the treating hospital and formally withdrawn from the study

Clinically Emergent Patient Condition

Should a matter be emergent (that is, requiring in-person assistance in less than 15 minutes), then 9-1-1 will be called and the patient will be returned to the hospital immediately. An example of an emergent patient condition is severe new-onset shortness of breath.

Clinically Urgent Patient Condition

Should a matter be urgent (that is, a condition that does not require assistance in under 15 minutes), the patient and/or nurse and/or physician may choose to communicate via phone or video (either of the 3 persons can initiate either medium). If this is unsuccessful, the nurse and/or physician will visit the

patient in their home. If there is no way to rectify the situation, then the patient will be transported to the hospital. An example of an urgent situation is new-onset non-severe pain.

Unsafe Home Situation

Should an unsafe home situation be discovered during a home hospital admission, the home hospital team will assess if said situation poses a harm or threat to either the participant or the home hospital personnel. If it does, and after an attempt to rectify said situation, the situation persists, then the home hospital team will end the study and return the patient to the hospital. An example of an unsafe home situations includes lack of basic sanitation.

Intoxication

Should intoxication occur in the home of a participant, the home hospital team will assess if said intoxication poses a harm or threat to either the participant or the home hospital personnel. If it does, and after an attempt to rectify said intoxication, the situation persists, then the home hospital team will end the study and return the patient to the hospital.

Neglect or child abuse

Should neglect or child abuse be observed, the home hospital team will act as mandatory reporters. The home hospital team, in coordination with the hotline team, will assess if said neglect or child abuse poses an immediate harm or threat to either the participant, children in the home, or the home hospital personnel. If it does, then the home hospital team will end the study and return the patient to the hospital.

Refusal of care

Should an enrolled subject refuse standard care at their home despite a clear discussion with the home hospital team, then the subject will be transferred back to the treating hospital and formally withdrawn from the study.

BIOSTATISTICAL ANALYSIS

Specific data variables being collected

Please see above.

Study endpoints

Study	Type	Primary	Secondary
Home hospital vs traditional hospital	Prospective intervention group, retrospective propensity-weighted evaluation	Cost of acute care episode	Cost of acute care episode and 30 days post-discharge<br>Length of stay<br>Unplanned readmission after index, y/n

			Number of adverse events<br>Picker experience questionnaire
Remote vs in-person home hospital physician	RCT, non-inferiority	Number of adverse events	Unplanned readmission after index, y/n<br>Picker experience questionnaire<br>Global experience score
Early transfer to home hospital vs traditional hospital	Prospective intervention group, retrospective propensity-weighted evaluation	Cost of acute care episode and 30 days post-discharge	Unplanned readmission after index, y/n<br>Picker experience questionnaire<br>Global experience score<br>Number of adverse events*
Paramedic + nurse care vs nurse care alone	Retrospective, non-inferiority, propensity-weighted evaluation	Number of adverse events	Unplanned readmission after index, y/n<br>Picker experience questionnaire<br>Global experience score<br>Personnel costs

*Non-inferiority for this outcome

Statistical methods

Home hospital vs traditional hospital

Quantitative

We plan to form 3 different control groups (with groups listed in order of preference):

1. Patients who were deemed eligible for home hospital, but the service was full (and they were not enrolled subsequently if the service was no longer full);
2. Patients who were deemed eligible for home hospital, but lived outside of the 7.5-mile catchment area; and
3. Patients who were admitted to the hospital.

For each control group, we will use propensity score weighting, adjusting for covariates of interest (see table above), baseline functional status, and DRG. We will be cognizant of over-adjustment depending on our final sample size for each group above. If possible, we will use only group 1 above, as it is the strongest. If we have insufficient control group sample size, we may choose to combine groups 1 and 2 above.

The primary outcome (hospitalization cost) will be assessed using a generalized linear model assuming a gamma distribution with a log link. We will use a multivariable regression approach for secondary

outcomes, using logistic regression for binary outcomes and non-transformed linear regression for normally distributed continuous outcomes as appropriate.

We *a priori* plan to perform a subgroup analysis that does not involve any outlier participants, should this occur. We also *a priori* plan subgroup analyses by diagnosis, by age group, by disposition, by location of admission (e.g., ED, floor, clinic, and home), and by daily activity level. We also *a priori* plan to analyze our outcomes with means, but also with median and interquartile ranges, which are less subject to outlier effects. We may choose to employ non-parametric tests of significance should our data be nonparametric.
We *a priori* plan to adjust our secondary analyses using the Benjamini-Hochberg method. We *a priori* choose a false-discovery rate of 0.20.

Qualitative
During and after the intervention period, we will interview a sample of patients and caregivers 7-30 days after discharge (irrespective of their experimental arm) regarding their opinions of their hospitalization. We will use this data to both iteratively improve home hospital and make detailed qualitative comparisons between home hospital and usual care.

In all cases, we will use semi-structured interview guides to ask questions regarding the hospitalization experience. Interviews will be recorded, transcribed, and then analyzed using NVivo qualitative analysis software. Drs. Schnipper and Levine will perform dual coding of all themes, and all differences will be discussed and reconciled. We will use an a priori analytic framework based on the Systems Engineering Initiative for Patient Safety Model (SEIPS), which describes processes and outcomes as products of the interactions among patients, technology and tools, the health care organization, the tasks at hand, and the physical environment.[24]

Remote vs In-Person Physician Care
In a randomized controlled non-inferiority trial, we will test an in-person daily physician visit ("in-person care;" control) vs a daily physician visit conducted remotely via video facilitated by a care team member ("remote care;" intervention) for acutely ill patients who are home hospitalized. The primary outcome (dependent variable) is number of adverse events during the acute episode of care (see "Summarizing Adverse Events" table below). The primary predictor (independent variable) is study arm (in-person care vs remote care). We will assess the non-inferiority of the primary outcome with a generalized linear model using Poisson distribution, given the primary outcome is count data. Although we have multiple covariates of interest, we are cognizant of over adjustment. We plan to adjust for age, gender, race/ethnicity, PRISMA-7,[25] education, and HOSPITAL score).[27] We may include additional covariates if groups have significant differences at baseline.

Because patients in the intervention arm will receive at least some of their intervention through a "change agent" (in this case, a Home Hospital team) shared with other participants, we need to account for this in the analysis. We will therefore cluster by Home Hospital physician using general estimating equations.

Other outcomes will be analyzed in a similar manner, using Poisson regression for other "count" outcomes (e.g., number of new or worsening signs or symptoms per patient, days at home since discharge), multivariable logistic regression for binary outcomes (e.g., 30-day readmission, ED visit, PCP

appointment within 7 or 14 days, ability to meet physical milestones after discharge), non-transformed linear regression for normally distributed continuous outcomes (e.g., quality of life scores), log-transformed linear regression for skewed continuous outcomes (e.g., cost), and Cox proportional hazards for time to event outcomes (e.g., time to readmission, time to ED visit). We will use multiple imputation for covariates where data are missing for more than 2% of patients. Lastly, we will perform a limited number of subgroup analyses to determine whether the intervention is more effective in certain patient populations than in others (i.e., effect modification), targeting inadequate health literacy and living alone, for example. We will use interaction terms (e.g., intervention*characteristic) to determine the statistical significance of effect modification.

Summarizing Adverse Events

Measure
Fall
Delirium
Potentially preventable VTE
New pressure ulcer
Thrombophlebitis at peripheral IV site
CAUTI
New Clostridium difficile
New MRSA
New arrhythmia
Hypokalemia
Acute kidney injury
Transfer back to hospital
Mortality (unplanned) during admission
Mortality (unplanned) 30-day post-discharge

Early transfer to home hospital vs traditional hospital
We plan to use the same statistical analysis as in the Standard Home Hospitalization methods noted above. However, we do anticipate requiring an additional variable that notes whether a patient was initially considered for home hospital care and was not eligible due to a full census, and then was later taken home when the home hospital service census allowed.

Paramedic Analysis
In a retrospective analysis, we plan to measure the difference in our slate of cost, quality, safety, and experience measures (above) between patients who were cared for when the paramedic was part of the care team vs patients who did not have an interaction with the paramedic. We plan to use the same statistical analysis as in the Standard Home Hospitalization methods noted above.

Artificial Intelligence Analysis
Please see separate attachment detailing artificial intelligence methods.

Continuous Glucose Monitoring Analysis
Concurrent glucose meter and sensor BG values will be compared by calculating both the mean difference (meter blood glucose minus sensor blood glucose) and mean absolute difference (absolute difference of the mean difference) between BG values, expressed both in mg/dL and as a percentage. A

Pearson correlation coefficient (r) will be calculated for the entire set of meter–sensor pairs. Finally, paired M-S values will be plotted on a Clarke Error Grid.

Power analysis

Standard Home Hospitalization

Our recent RCT data suggest that at 90% power, we will require 38 patients in each arm. However, we also have prespecified a smallest important difference of $1000, for which we will need 144 patients in each arm at 90% power.

For 30-day readmission, we will require 110 patients in each arm at 90% power (23% vs 7%). If the effect size is indeed smaller, for example 23% vs 15%, we will need 528 patients in each arm. However, we will not be able to power around a smallest important difference of 3%, for which we would need 4006 patients in each arm.

For percentage with a safety event, we will require 641 patients in each arm at 90% power and 493 patients in each arm at 80% power (15% vs 9%).

Remote vs In-Person Care Analysis

During our prior RCT of home hospital, patients experienced a mean of 0.09 (SD 0.29) adverse events during their home hospital admission. We believe the smallest clinically meaningful treatment difference would be 0.1 adverse events (i.e., from 0.09 to 0.19 adverse events per patient). In this non-inferiority trial (alpha=0.025, power 80%, non-inferiority limit 0.1 events), we would require 210 patients (105 in each arm). Because of clustering, the actual required sample size will be larger. With 5 home hospital physicians (rho 0.01), we require 260 patients (130 in each arm). We consistently enroll 25 patients monthly, leaving us with sufficient power after about 11 months.

We plan an interim futility analysis. After 130 patients have completed the remote vs in-person care study, we will calculate the difference in adverse event rates between the two arms. If the difference is 0.10 or higher (with the higher rate in the intervention arm), then we will stop the trial for futility. This rule will reduce the power about 2%.

Early Transfer

To calculate sample size, we used simulation in the PASS program (NCSS LLC, Kaysville, UT) comparing gamma distributed costs, with baseline data based on the home hospital RCT. With 324 patients in each arm of the study, even accounting for intraclass correlation by Home Hospital physician (intraclass correlation coefficient 0.01, cluster size 65), we will have 82% power to detect a relative decrease of 21% in total costs of care, smaller than the 27% decrease seen in this outcome in the Home Hospital RCT and close to the smallest reduction that would be considered clinically important**.** This sample size will also provide 80% power to detect a 43% reduction in readmission (again smaller than that seen in the Home Hospital RCT), and 81% power to detect non-inferiority for adverse events (assuming a baseline of 0.15 events per patient).

Paramedic Analysis
Similar power calculations as those in the standard home hospital and remote vs in-person care analysis apply.

RISKS AND DISCOMFORTS

Uncommon: Complications of surgical and non-surgical procedures

Standard hospital procedures carry small risks and discomforts. For example, venipuncture can be painful and can lead to thrombophlebitis, but this is uncommon and readily rectifiable.

Uncommon: Device complications/malfunctions

IV pumps, vital signs monitoring patches, and the other devices used during home hospital uncommonly have malfunctions.

Uncommon: Radiation risks

Patients can receive radiography while admitted to home hospital, much the same as when they are admitted in the hospital. We do not anticipate that home hospital will in any way change the prevailing risk of radiation.

Common (but unchanged from usual care): Drug side effects and toxicities

Drug side effects and toxicities do occur during inpatient medicine, despite following best practices and evidence based medicine. We do not anticipate that home hospital will in any way change the prevailing rate of drug side effects and toxicities compared to usual care. For example, acute kidney injury can occur in patients receiving antibiotics, even when correct dosing occurs. Home hospital is equipped to monitor and respond to the common drug side effects and toxicities much the same as the standard of care (monitoring, diagnostics, fluids, etc).

Common (but improved from usual care): Psychosocial risks

Admission to the hospital can be a psychosocially difficult event, particularly for a senior. We believe home hospital will alleviate these typical risks. Home hospital is equipped to assist patients with psychosocial stressors through improved staffing ratios and a social worker.

POTENTIAL BENEFITS

Potential benefits to participating individuals

- Remain in their home despite acute illness
  - Eat their own culturally concordant food
  - Sleep in familiar surroundings
  - Maximize time and interactions with their family and friends
- Improved health outcomes (we anticipate these from previous literature)
  - Reduced length of stay
  - Reduced complications while admitted

        - Less delirium
        - Fewer falls
        - Fewer health care associated infections
        - Decreases reduction in functional status
    - Improved patient experience
- Clinical improvements to the standard of care
    - Point of care blood diagnostics (results at the bedside in <5 minutes);
    - Minimally invasive continuous vital signs, telemetry, and activity tracking, including clinical deterioration algorithms, steps taken, and sleep quality;
    - On-demand 24/7 clinician virtual video visits;
    - 12 to 1 patient to MD ratio, compared to typical 16 to 1;
    - Ambulatory/portable infusion pumps that can be worn on the hip;
    - Optional access to a personal home health aide, community health worker, and community paramedic
- Improved transitions of care
    - Ability to coach patients on their post-discharge care plan in the appropriate environment, with caregivers available, and with adequate time for teaching given provider to patient ratio

Potential benefits to society

- New evidence-based care paradigm for acute care hospitalization.
- Lower total medical expenditure. Allows for redirection of resources to areas in need.
- Might eventually lead to reduction in total hospitals in the U.S.
- Provide needed randomized controlled data on the home hospital intervention to inform payment methods moving forward.

MONITORING AND QUALITY ASSURANCE

Independent monitoring of source data

The study's research assistant will collect all data denoted above. The PI and co-PI will monitor the source data throughout the project.

Safety monitoring

If any of the following concerning safety events occur, a blinded monitoring committee will review the event to determine attribution to the intervention: fall, medication error, DVT/PE, mortality during admission, transfer back to hospital (committee cannot be blinded for this endpoint). If any of these events are felt to be due to the home hospital intervention, they will be reported to the IRB with recommendations for appropriate actions to be taken. Decisions to modify the protocol or suspend the study will be made jointly by the study investigators, monitoring committee, and the IRB.

Outcomes monitoring

The physician and RNs will daily review quality and safety data as part of a rapid logistics improvement process.

Weekly, all physicians and RNs will huddle to review quality and safety data as part of a rapid logistics improvement process.

At 3 weeks into the home hospital clinical service, a blinded monitoring committee will review outcomes data and make recommendations to the study team.

Adverse event reporting guidelines

All adverse events denoted above under safety monitoring will be reported to the monitoring committee.

APPENDIX

Selected Safety data to-date

	n (%)
Adverse Event[a]	Home (n=203)
30-day unplanned readmission	15 (7)
Transfer back to hospital	5 (2)
Delirium	5 (2)
New DVT/PE	0
New pressure ulcer	2 (1)
Thrombophlebitis at peripheral IV site	4 (2)
Catheter-associated urinary tract infection	0
New Clostridium difficile	0
New MRSA	0
New arrhythmia in heart failure[b]	0
Mortality during hospitalization	0

[a] None of these adverse events were deemed secondary to the intervention.

Risk Scores

CURB 65 – 30-day mortality prediction

- Cohort: 1068pts of CAP in the UK, New Zealand, and the Netherlands in 1998-1999
- 30-day mortality by points
    - score 0, 0.7%
    - score 1, 3.2%
    - score 2, 3%
    - score 3, 17%
    - score 4, 41.5%
    - score 5, 57%

No of features	Sensitivity (%)	Specificity (%)	PPV (%)	NPV (%)
>0	100	0	9.6	NA
>1	98.6	21.0	11.7	99.3
>2	92.8	49.2	16.2	98.5
>3	68.1	74.9	22.4	95.7
>4	39.1	93.1	37.5	93.5
>5	1.4	99.1	14.3	90.4

SMRTCO – chance of intensive respiratory or vasopressor support (IRVS)

- Cohort: 5 databases (7464 patients), 1991-2001
- IRVS by points
    - Score 0, ~0%
    - Score 1, 5%
    - Score 2, 10%

- Score 3, 17%
- Score >=4, 33%

Nonelectrocardiographic categorizations for artificial intelligence

- Of note, several additional categorizations will be built during this project. This is a preliminary list.Loss of consciousness
- Delirium
- Cough
- Obstructive sleep apnea
- Sleep-disordered breathing
- Inappropriate response to activity
- Sepsis
- Stress

Study Staff

- David Levine, MD MPH MA, is an associate physician the division of general internal medicine and primary care at Brigham and Women's Hospital and instructor in medicine at Harvard Medical School. He has expertise in design, implementation, and evaluation of innovative and disruptive medical programs with experience in house calls, digital health, and community health worker programs. Currently leading projects on national quality measurement, disparities in quality, virtual visits, seniors' use of digital health, and house calls. As principle investigator of this project, his role is in design, implementation, and evaluation.
- Jeffrey Schnipper, MD MPH, has conducted research since 2002 aimed at improving health information technology, predicting hospital readmission, improving medication safety during transitions in care, and improving discharge. He has over 100 peer-reviewed publications, including a dual-site randomized controlled trial of an electronic medication reconciliation tool and process redesign on patient safety, generally regarded as the most rigorous study to date of electronic medication reconciliation tools. More recently, his group completed a two-site randomized controlled trial of pharmacist counseling and follow-up on cardiac patients with low health literacy (the PILL-CVD study). He was the principal investigator of the Multi-Center Medication Reconciliation Quality Improvement Study (MARQUIS), an AHRQ-funded study at five US hospitals to design and implement an evidence-based toolkit to improve medication reconciliation and is now the PI of an AHRQ-funded study to continue that work at 18 additional hospitals. Dr. Schnipper's recent PCORI-funded readmission avoidance project involved community health workers. He also has expertise in decision support, electronic dashboards, and the novel use of patient portals.
- 3 research assistants – TBD

REFERENCES

1. Hung WW, Ross JS, Farber J, Siu AL. Evaluation of the Mobile Acute Care of the Elderly (MACE) service. *JAMA Intern Med*. 2013;173(11):990-996. doi:10.1001/jamainternmed.2013.478.

2. Fong TG, Tulebaev SR, Inouye SK. Delirium in elderly adults: diagnosis, prevention and treatment. *Nat Rev Neurol*. 2009;5(4):210-220. doi:10.1038/nrneurol.2009.24.

3. *2014 National and State Healthcare-Associated Infections Progress Report*.; 2016. http://www.cdc.gov/hai/surveillance/progress-report/index.html. Accessed April 19, 2016.

4. Counsell SR, Holder CM, Liebenauer LL, et al. Effects of a Multicomponent Intervention on Functional Outcomes and Process of Care in Hospitalized Older Patients: A Randomized Controlled Trial of Acute Care for Elders (ACE) in a Community Hospital. *J Am Geriatr Soc*. 2000;48(12):1572-1581. doi:10.1111/j.1532-5415.2000.tb03866.x.

5. Caplan GA, Ward JA, Brennan NJ, Coconis J, Board N, Brown A. Hospital in the home: a randomised controlled trial. *Med J Aust*. 1999;170(4):156-160. http://www.ncbi.nlm.nih.gov/pubmed/10078179. Accessed September 19, 2017.

6. Board N, Brennan N, Caplan GA. A randomised controlled trial of the costs of hospital as compared with hospital in the home for acute medical patients. *Aust N Z J Public Health*. 2000;24(3):305-311. http://www.ncbi.nlm.nih.gov/pubmed/10937409. Accessed February 17, 2017.

7. Montalto M. The 500-bed hospital that isn't there: the Victorian Department of Health review of the Hospital in the Home program. *Med J Aust*. 2010;193(10):598-601. http://www.ncbi.nlm.nih.gov/pubmed/21077817. Accessed February 19, 2016.

8. Montalto M, Lui B, Mullins A, Woodmason K. Medically-managed Hospital in the Home: 7 year study of mortality and unplanned interruption. *Aust Health Rev*. 2010;34(3):269-275. doi:10.1071/AH09771.

9. Tibaldi V, Isaia G, Scarafiotti C, et al. Hospital at Home for Elderly Patients With Acute Decompensation of Chronic Heart Failure. *Arch Intern Med*. 2009;169(17):1569-1575. doi:10.1001/archinternmed.2009.267.

10. Hernandez C, Casas A, Escarrabill J, et al. Home hospitalisation of exacerbated chronic obstructive pulmonary disease patients. *Eur Respir J*. 2003;21(1):58-67. http://www.ncbi.nlm.nih.gov/pubmed/12570110. Accessed October 30, 2017.

11. Leff B, Burton L, Mader SL, et al. Hospital at home: feasibility and outcomes of a program to provide hospital-level care at home for acutely ill older patients. *Ann Intern Med*. 2005;143(11):798-808. http://www.ncbi.nlm.nih.gov/pubmed/16330791. Accessed February 15, 2016.

12. Cryer L, Shannon SB, Van Amsterdam M, Leff B. Costs for "hospital at home" patients were 19 percent lower, with equal or better outcomes compared to similar inpatients. *Health Aff (Millwood)*. 2012;31(6):1237-1243. doi:10.1377/hlthaff.2011.1132.

13. Levine DM, Ouchi K, Blanchfield B, et al. Hospital-Level Care at Home for Acutely Ill Adults: a Pilot Randomized Controlled Trial. *J Gen Intern Med*. February 2018. doi:10.1007/s11606-018-4307-z.

14. Rabin RF, Jennings JM, Campbell JC, Bair-Merritt MH. Intimate partner violence screening tools: a systematic review. *Am J Prev Med*. 2009;36(5):439-445.e4. doi:10.1016/j.amepre.2009.01.024.

15. Chalmers JD, Singanayagam A, Hill AT. Systolic blood pressure is superior to other haemodynamic predictors of outcome in community acquired pneumonia. *Thorax*. 2008;63(8):698-702. doi:10.1136/thx.2008.095562.

16. Charles PGP, Wolfe R, Whitby M, et al. SMART-COP: a tool for predicting the need for intensive respiratory or vasopressor support in community-acquired pneumonia. *Clin Infect Dis*. 2008;47(3):375-384. doi:10.1086/589754.

17. Peterson PN, Rumsfeld JS, Liang L, et al. A validated risk score for in-hospital mortality in patients with heart failure from the American Heart Association get with the guidelines program. *Circ Cardiovasc Qual Outcomes*. 2010;3(1):25-32. doi:10.1161/CIRCOUTCOMES.109.854877.

18. Fonarow GC, Adams KF, Abraham WT, Yancy CW, Boscardin WJ. Risk stratification for in-hospital mortality in acutely decompensated heart failure: classification and regression tree analysis. *JAMA*. 2005;293(5):572-580. doi:10.1001/jama.293.5.572.

19. Singer M, Deutschman CS, Seymour CW, et al. The Third International Consensus Definitions for Sepsis and Septic Shock (Sepsis-3). *JAMA*. 2016;315(8):801. doi:10.1001/jama.2016.0287.

20. Wallia A, Umpierrez GE, Rushakoff RJ, et al. Consensus Statement on Inpatient Use of Continuous Glucose Monitoring. *J Diabetes Sci Technol*. 2017;11(5):1036-1044. doi:10.1177/1932296817706151.

21. Schierenbeck F, Franco-Cereceda A, Liska J. Accuracy of 2 Different Continuous Glucose Monitoring Systems in Patients Undergoing Cardiac Surgery. *J Diabetes Sci Technol*. 2017;11(1):108-116. doi:10.1177/1932296816651632.

22. Boom DT, Sechterberger MK, Rijkenberg S, et al. Insulin treatment guided by subcutaneous continuous glucose monitoring compared to frequent point-of-care measurement in critically ill patients: a randomized controlled trial. *Crit Care*. 2014;18(4):453. doi:10.1186/s13054-014-0453-9.

23. Smith SW, Walsh B, Grauer K, et al. A deep neural network learning algorithm outperforms a conventional algorithm for emergency department electrocardiogram interpretation. *J Electrocardiol*. 2019;52:88-95. doi:10.1016/J.JELECTROCARD.2018.11.013.

24. Carayon P, Schoofs Hundt A, Karsh B-T, et al. Work system design for patient safety: the SEIPS model. *Qual Saf Health Care*. 2006;15 Suppl 1:i50-8. doi:10.1136/qshc.2005.015842.

25. Raîche M, Hébert R, Dubois M-F. PRISMA-7: a case-finding tool to identify older adults with moderate to severe disabilities. *Arch Gerontol Geriatr*. 2008;47(1):9-18. doi:10.1016/j.archger.2007.06.004.

26. Galvin JE, Roe CM, Powlishta KK, et al. The AD8: a brief informant interview to detect dementia. *Neurology*. 2005;65(4):559-564. doi:10.1212/01.wnl.0000172958.95282.2a.

27. Donzé J, Aujesky D, Williams D, Schnipper JL. Potentially Avoidable 30-Day Hospital Readmissions in Medical Patients. *JAMA Intern Med*. 2013;173(8):632. doi:10.1001/jamainternmed.2013.3023.
