# Supplementary material for: Remote vs In-home Physician Visits for Hospital-Level Care at Home: A Randomized Clinical Trial
Source: JAMA Netw Open. 2022 Aug 30;5(8):e2229067. doi: 10.1001/jamanetworkopen.2022.29067 (PMC9428739; doi:10.1001/jamanetworkopen.2022.29067)
Supplement: Supplement 2. — eAppendix. Detailed Inclusion and Exclusion Criteria eTable 1. Characteristics of Patients Who Enrolled and Declined eTable 2. Characteristics of Patients Who Required In-home Visits vs Those Who Did Not, Among the Remote Group [file jamanetwopen-e2229067-s002.pdf]

# Supplementary Online Content

Levine DM, Paz M, Burke K, et al. Remote vs in-home physician visits for hospital-level care at home: a randomized clinical trial. *JAMA Netw Open*. 2022;5(8):e2229067. doi:10.1001/jamanetworkopen.2022.29067

**eAppendix**. Detailed Inclusion and Exclusion Criteria

**eTable 1**. Characteristics of Patients Who Enrolled and Declined

**eTable 2**. Characteristics of Patients Who Required In-home Visits vs Those Who Did Not, Among the Remote Group

This supplementary material has been provided by the authors to give readers additional information about their work.

© 2022 Levine DM et al. *JAMA Network Open.*

**eAppendix**. Detailed Inclusion and Exclusion Criteria

*Inclusion*

- Clinical
  - >=18 years old
  - Primary or possible diagnosis of any infection, heart failure exacerbation, chronic obstructive pulmonary disease (COPD) exacerbation, asthma exacerbation, chronic kidney disease requiring diuresis, diabetes and its complications, gout exacerbation, hypertensive urgency, previously diagnosed atrial fibrillation with rapid ventricular response, anticoagulation needs (e.g., venous thromboembolism), or a patient at the end of life who desired only medical management

*Exclusion*

- Social
  - Undomiciled
  - No working heat (October-April), no working air conditioning if forecast > 80°F (June-September), or no running water
  - On methadone requiring daily pickup of medication
  - In police custody
  - Resides in facility that provides on-site medical care (e.g., skilled nursing facility)
  - Domestic violence screen positive[1]
- Clinical
  - Acute delirium, as determined by the Confusion Assessment Method
  - Cannot establish peripheral access in emergency department
  - Secondary condition: active non-melanoma/prostate cancer, end-stage renal disease, acute myocardial infarction, acute cerebral vascular accident, acute hemorrhage
  - Primary diagnosis requires multiple or routine administrations of controlled substances for pain control
  - Cannot independently ambulate to bedside commode
  - As deemed by on-call MD, patient likely to require any of the following procedures: computed tomography, magnetic resonance imaging, endoscopic procedure, blood transfusion, cardiac stress test, or surgery
  - For pneumonia:
    - Most recent CURB65 > 3[10]
    - Most recent SMRTCO > 2[11]
    - Absence of clear infiltrate on imaging
    - Cavitary lesion on imaging
    - Pulmonary effusion of unknown etiology
    - $O_2$ saturation < 90% despite 5L $O_2$
  - For heart failure:
    - Has a left ventricular assist device
    - GWTG-HF[12] (>10% in-hospital mortality) or ADHERE[13] (high risk or intermediate risk 1)*
    - Severe pulmonary hypertension
  - For complicated urinary tract infection:
    - Absence of pyuria
    - Most recent qSOFA > 1[14]

© 2022 Levine DM et al. *JAMA Network Open.*

- For other infection
    - Most recent qSOFA > 1[14]
- For COPD
    - BAP-65 score > 3
- For asthma
    - Peak expiratory flow < 50% of normal: exercise caution
- For diabetes and its complications
    - Requires IV insulin
- For hypertensive urgency
    - Systolic blood pressure > 190 mmHg
    - Evidence of end-organ damage
- For atrial fibrillation with rapid ventricular response
    - Likely to require cardioversion
    - New atrial fibrillation with rapid ventricular response
    - Unstable blood pressure, respiratory rate, or oxygenation
    - Despite IV beta and/or calcium channel blockade in the emergency department, HR remains > 125 and SBP remains different than baseline
    - Less than 1 hour of time has elapsed with HR < 125 and SBP similar or higher than baseline

© 2022 Levine DM et al. *JAMA Network Open.*

**eTable 1**. Characteristics of Patients Who Enrolled and Declined

| | **Enrolled** (n=172) | **Declined** (n=105) |
|---|---|---|
| **Age**, y, mean (95% CI) | 69.3 (66.6,72.0) | 66.4 (62.8, 70.0) |
| **Female**, n (%) | 97 (56.4) | 70 (66.7) |
| **Race/ethnicity**, n (%) | | |
| White | 77 (45.0) | 56 (53.3) |
| Black | 33 (19.3) | 28 (26.7) |
| Latin@ | 39 (22.8) | 9 (8.6) |
| Other | 22 (12.9) | 12 (11.4) |
| **Partner status**, n (%) | | |
| Partnered | 81 (47.3) | 34 (33.3) |
| Divorced | 15 (8.8) | 14 (13.7) |
| Widowed | 18 (10.5) | 17 (16.7) |
| Single, never partnered | 56 (32.8) | 36 (35.3) |
| Other | 1 (0.6) | 1 (1) |
| **Primary language**, n (%) | | |
| English | 124 (72.1) | 90 (85.7) |
| Spanish | 43 (25.0) | 11 (10.5) |
| Other | 2 (1.2) | 4 (3.8) |
| **Insurance**, n (%) | | |
| Private | 51 (29.7) | 26 (24.8) |
| Medicare | 93 (54.1) | 45 (42.9) |
| Medicaid | 7 (4.1) | 14 (13.3) |
| Medicare+Medicaid | 20 (11.6) | 17 (16.2) |
| None | 1 (0.6) | 3 (2.9) |

Note: Our IRB only allowed for collection of the above variables for patients who declined enrollment.

© 2022 Levine DM et al. *JAMA Network Open.*

**eTable 2**. Characteristics of Patients Who Required In-Home Visits vs Those Who Did Not, Among the Remote Group

| | **Required In-Home Visit** (n=16) | **Did Not Require In-Home Visit** (n=68) |
|---|---|---|
| **Age**, y, mean (95% CI) | 69.7 (59.9,79.5) | 72.7 (68.8,76.6) |
| **Female**, n (%) | 9 (56.3) | 42 (61.8) |
| **Race/ethnicity**, n (%) | | |
| White | 5 (31.3) | 27 (39.7) |
| Black | 3 (18.8) | 13 (19.1) |
| Latin@ | 4 (25) | 17 (25) |
| Other | 4 (25) | 11 (16.2) |
| **Partner status**, n (%) | | |
| Partnered | 7 (43.8) | 28 (41.2) |
| Divorced | 3 (18.8) | 6 (8.8) |
| Widowed | 2 (12.5) | 9 (13.2) |
| Single, never partnered | 4 (25) | 25 (36.8) |
| Other | - | - |
| **Primary language**, n (%) | | |
| English | 8 (50) | 49 (72.1) |
| Spanish | 7 (43.8) | 16 (23.5) |
| Other | 1 (6.3) | 3 (4.4) |
| **Insurance**, n (%) | | |
| Private | 5 (31.3) | 13 (19.1) |
| Medicare | 9 (56.3) | 44 (64.7) |
| Medicaid | 1 (6.3) | 3 (4.4) |
| Medicare+Medicaid | 1 (6.3) | 8 (11.8) |
| None | - | - |
| **Education**, n (%) | | |
| <High school | 5 (41.7) | 13 (25) |
| High school | - | 12 (23.1) |
| <4-year college | 4 (33.3) | 11 (21.2) |
| 4-year college | - | 10 (19.2) |
| >4-year college | 3 (25) | 6 (11.5) |
| **Employment**, n (%) | | |
| Employed | 2 (15.4) | 11 (16.7) |
| Unemployed | 4 (30.8) | 10 (15.2) |
| Retired | 7 (53.9) | 45 (68.2) |
| **Cigarette smoking**, n (%) | | |
| Never | 7 (43.8) | 35 (52.2) |
| Current | 1 (6.3) | 3 (4.5) |
| Prior | 8 (50) | 29 (43.3) |
| **Lives alone**, n (%) | 4 (25) | 18 (26.5) |
| **PRISMA** (0-7), mean (95% CI) | 3.8 (2.3,5.3) | 3.3 (2.9,3.7) |

© 2022 Levine DM et al. *JAMA Network Open.*

| | | |
|---|---|---|
| **Ascertain dementia-8** (0-8), mean (95% CI) | 1.3 (0,3) | 1.7 (0.9,2.4) |
| **BHLS** (4-20), mean (95% CI)[a] | 14.9 (10.6,19.2) | 17.6 (16.4,18.8) |
| **Comorbidity count**, mean (95% CI)[b] | 3.2 (2,4.4) | 4.1 (3.5,4.6) |
| **Code status: Full code**, n (%)[c] | 12 (75) | 59 (88.1) |
| **EuroQol VAS** (0-100), mean (95% CI) | 62.2 (41.6,82.8) | 54.7 (47.9,61.5) |
| **ADLs** on admission (0-6), mean (95% CI) | 4.3 (2.4,6.1) | 5.2 (4.7,5.7) |
| **IADLs** on admission (0-8), mean (95% CI) | 3.9 (2,5.8) | 5.5 (4.8,6.2) |
| **PHQ-2** (0-6), mean (95% CI) | 0.7 (0,1.4) | 1.1 (0.6,1.6) |
| **Hospital admission in last 6 months**, n (%) | 7 (43.8) | 22 (32.4) |
| **Emergency department visit in last 6 months**, n (%) | 3 (18.8) | 24 (35.3) |
| **Diagnosis**, n (%)[d] | | |
| Infection | | |
| Pneumonia | 1 (6.3) | 13 (19.1) |
| Skin/soft tissue infection | 1 (6.3) | 8 (11.8) |
| Complicated UTI/pyelonephritis | 5 (31.3) | 12 (17.7) |
| Other infection | 3 (18.8) | 8 (11.8) |
| Heart failure | 1 (6.3) | 8 (11.8) |
| Airway disease | | |
| Asthma | 1 (6.3) | 7 (10.3) |
| COPD | 2 (12.5) | 6 (8.8) |
| Other[e] | 2 (12.5) | 6 (8.8) |

[a]: Brief Health Literacy Screener, 4-12: limited; 13-16: marginal; 17-20: adequate

[b]: Count of patient's chronic comorbidities

[c]: Patients who chose resuscitation and intubation

[d]: Diagnoses were block randomized at the level of infection, heart failure, airway disease, and other.

[e]: Other diagnoses such as atrial fibrillation with rapid ventricular response, diabetes mellitus, pulmonary embolism, and others (see the **Supplement** for complete list and criteria).

**Abbreviations**: ADLs, activities of daily living; BHLS, Brief Health Literacy Screener; CI, confidence interval; COPD, chronic obstructive pulmonary disease; IADLs, instrumental activities of daily living; PHQ-2, Patient Health Quesitonnaire-2 (measure of depression); PRISMA, Program of Research to Integrate Services for the Maintenance of Autonomy (measure of frailty; scores >2 indicate frailty); PROMIS, Patient-Reported Outcomes Measurement Information System; UTI, urinary tract infection; VAS, Visual Analogue Scale

© 2022 Levine DM et al. *JAMA Network Open.*
