# Supplementary material for: Remote vs In-home Physician Visits for Hospital-Level Care at Home: A Randomized Clinical Trial
Source: JAMA Netw Open. 2022 Aug 30;5(8):e2229067. doi: 10.1001/jamanetworkopen.2022.29067 (PMC9428739; doi:10.1001/jamanetworkopen.2022.29067)
Supplement: Supplement 3. — Data Sharing Statement [file jamanetwopen-e2229067-s003.pdf]

Data Sharing Statement

Levine. Remote vs In-Home Physician Visits for Hospital-Level Care at Home. *JAMA Netw Open*. Published August 30, 2022. doi:10.1001/jamanetworkopen.2022.29067

Data
Data available: No
